# Indole‐3‐Lactic Acid Inhibits Doxorubicin‐Induced Ferroptosis Through Activating Aryl Hydrocarbon Receptor/Nrf2 Signalling Pathway

**DOI:** 10.1111/jcmm.70358

**Published:** 2025-01-23

**Authors:** Jiangfang Lian, Hui Lin, Zuoquan Zhong, Yongfei Song, Xian Shao, Jiedong Zhou, Lili Xu, Zhenzhu Sun, Yongyi Yang, Jufang Chi, Ping Wang, Liping Meng

**Affiliations:** ^1^ Department of Cardiology The Affiliated Lihuili Hospital of Ningbo University Zhejiang China; ^2^ Central Laboratory of Medicine Shaoxing People's Hospital Shaoxing China; ^3^ College of Medicine Shaoxing University Shaoxing China; ^4^ Department of Cardiology, Enze Medical Research Center Taizhou Hospital Affiliated to Wenzhou Medical University Linhai China; ^5^ Department of Gynaecology, The First Affiliated Hospital, College of Medicine Zhejiang University Hangzhou China; ^6^ Department of Cardiology, Zhuji People's Hospital of Zhejiang Province Zhuji Affiliated Hospital of Wenzhou Medical University Zhuji China; ^7^ Department of Cardiology Shaoxing People's Hospital, Shaoxing Hospital, Zhejiang University School of Medicine Shaoxing China

**Keywords:** cardiotoxicity, doxorubicin, ferroptosis, gut microbiota, indole‐3‐lactic acid

## Abstract

The clinical application of doxorubicin (DOX) is limited due to its cardiotoxicity, which is primarily attributed to its interaction with iron in mitochondria, leading to lipid peroxidation and myocardial ferroptosis. This study aimed to investigate the role of the gut microbiota‐derived metabolite, indole‐3‐lactic acid (ILA), in mitigating DOX‐induced cardiotoxicity (DIC). Cardiac function, pathological changes, and myocardial ferroptosis were assessed in vivo. The cardioprotective effects and mechanisms of ILA were explored using multi‐omics approaches, including single‐nucleus RNA sequencing (snRNA‐seq) and bulk RNA‐seq, and were further validated in Nrf2 knockout mice. The findings revealed that DOX treatment disrupted gut microbiota, significantly reducing the levels of the tryptophan metabolite ILA. In DIC models, ILA supplementation markedly improved cardiac function, reduced collagen deposition, and mitigated cardiac atrophy. The bulk and snRNA‐seq analyses indicated that myocardial ferroptosis played a crucial role in the cardioprotective effects of ILA. Experimental data demonstrated that ILA decreased DOX‐induced ferroptosis in both DIC mice and DOX‐treated H9C2 cells, evidenced by restoration of GPX4 and SLC7A11 levels and reduction of ACSL4. Mechanistically, ILA functions as a ligand for the aryl hydrocarbon receptor (AhR), leading to the upregulation of Nrf2 expression. The protective effects of ILA against ferroptosis were abolished by silencing AhR. Moreover, the beneficial effects of ILA on DIC were eliminated in Nrf2‐deficient mice. In conclusion, ILA exerts therapeutic effects against DIC by inhibiting ferroptosis through activation of the AhR/Nrf2 signalling pathway. Identifying the cardioprotective role of the microbial metabolite ILA could offer viable therapeutic strategies for DIC.

**Abbreviations**
AhR: activating aryl hydrocarbon receptor
DIC: DOX‐induced cardiotoxicity
DOX: doxorubicin
GPX4: glutathione peroxidase 4
HO‐1: heme oxygenese‐1
IAA: indole‐3‐acetic acid
ILA: indole‐3‐lactic acid
IPA: indole‐3‐propionic acid
Keap1: Kelch‐like ECH‐associated protein 1
NRF2: nuclear factor erythroid 2‐related factor 2
SLC7A11: solute carrier family 7 member 11
snRNA‐seq: single‐nucleus RNA sequencing

## 1 Introduction

Doxorubicin (DOX) is a widely used chemotherapeutic agent with demonstrated efficacy against various types of cancers. However, cardiotoxicity is a frequent and often lethal complication of DOX‐based chemotherapy [1]. It typically leads to progressive systolic dysfunction of the left ventricle and the subsequent development of heart failure with lifelong consequences [2]. Given the increasing burden of cancer and heart failure in the aging population, the development of effective cardioprotective strategies is necessary to minimise the impact of DOX‐induced cardiotoxicity (DIC). Dexrazoxane is currently the only drug approved for the treatment of DIC. However, its use has been associated with reduced antitumor efficacy of doxorubicin and an increased risk of secondary malignancies. Consequently, the FDA has restricted its use to patients with metastatic breast cancer who are expected to receive cumulative doxorubicin doses exceeding 300 mg/m^2^ [3]. Several mechanisms, including oxidative stress, impaired mitochondrial function, disruption of Ca^2+^ homeostasis, and apoptosis [4], have been reported to be involved in DIC. Recent studies have focused on ferroptosis during DIC development [5]. Ferroptosis is a novel form of programmed cell death characterised by the iron‐dependent accumulation of lipid peroxides and reduced activity of the antioxidant enzyme glutathione peroxidase‐4 (GPX4) [6]. Inhibition of ferroptosis using ferrostatin‐1 and other agents has been shown to effectively prevent DIC [7, 8]. Therefore, finding an effective method to inhibit ferroptosis may represent a potential therapeutic strategy against DIC.

The use of a blend of antioxidants has shown the potential to reduce DIC. Our previous study showed that yellow wine polyphenolic compounds [9], dihydromyricetin [10] and fucoidan [11] alleviate DOX‐induced cardiomyocyte apoptosis and pyroptosis. However, few studies have focused on the endogenous metabolic changes upon DOX treatment. Gut microbiota‐host interactions are closely linked to DIC [12] and studies have shown that DOX causes composition imbalance and functional changes in the gut microbiota and their metabolites [13], which may result from disruption of the physical intestinal barrier and small intestinal mucositis [14]. DOX‐induced damage to the intestine leads to an imbalance in the gut microbiota [15]. We have also reported reduced bifidobacteria and dysbiosis of tryptophan metabolism in DOX‐treated rats [16]. Our study confirmed that faecal microbiota transplantation alters the gut microbiota and serum metabolites of recipient mice to protect against DIC, and correlation analysis indicated a close association between serum tryptophan metabolites and cardiac function [17]. Bifidobacterial culture supernatants exclusively produce indole‐3‐lactic acid (ILA) among tryptophan metabolites, whereas indole‐3‐propionic acid, indole‐3‐acetic acid and indole‐3‐aldehyde are not produced [18]. ILA ameliorates tumorigenesis via epigenetic regulation of cell immunity [19] and has been shown to possess anti‐inflammatory properties in various organs [20, 21]; however, its potential role in DOX‐induced cardiomyocyte ferroptosis and the underlying mechanisms remain unclear.

Therefore, this study aimed to explore the protective role of ILA against DOX‐induced ferroptosis in cardiomyocytes. To the best of our knowledge, this is the first investigation to examine the healing potential of tryptophan metabolites on DIC both in vivo and in vitro, which offers new perspectives on the function of the gut microbiota and their byproduct, ILA, in treating DIC.

## 2 Materials and Methods

### 2.1 In Vivo Study and Treatment

A total of 36 male C57BL/6 mice (6–8 weeks, 22–24 g) were obtained from the Zhejiang Chinese Medical University (Zhejiang, China). In addition, wild‐type (WT), Nrf2‐knockout (Nrf2^−/−^, Nrf2‐KO, Strain NO. T002625) were purchased from GemPharmatech Co. Ltd. (NanjingJiangsu, China), and six WT and Nrf2^−/−^ mice per group were treated with or without ILA (24 mice in total). All mice were maintained under a 12‐h light and dark cycle at a temperature of 25°C ± 2°C, and they were provided with a sufficient standard animal diet and drinking water. Mice were randomised into six groups (six mice per group). The DOX group received an intravenous tail injection of DOX (5 mg/kg once per week; MedChemExpress, Shanghai, China) for 4 weeks to achieve an accumulated total dose of 20 mg/kg. Mice in the ILA group were administered 12.5, 25 or 50 mg/kg ILA (Cat. I157602; > 98.0%(HPLC), Aladdin, Shanghai, China) per mouse per day for 4 weeks by gavage. ILA was dissolved by sterile saline and NaOH (1 N, pH = 7.2–7.4). The DOX + ILA group received DOX as well as ILA. Mice in the dexrazoxane group were intraperitoneally administered 60 mg/kg dexrazoxane solution per week 2 h before DOX treatment [22]. The control group was administered an equal volume of sterile saline. The selection of suitable dosages and methods for dissolving DOX and ILA was guided by previous studies [23, 24, 25, 26]. At the end of the fourth week, the mice were euthanized by CO_2_‐induced euthanasia, and heart, blood and stool samples were collected and stored. All procedures were approved by the Animal Care and Use Committee of Shaoxing People's Hospital (No. 2020‐002).

### 2.2 Echocardiography

The mice were anaesthetised with 2% isoflurane (RWD Life Science Co. Ltd., China) for cardiac function analysis (heart rate stabilised at 400–500 beats/min). The Vevo 2100 Imaging System (version 1.3.0) from VisualSonics was used to perform transthoracic echocardiography. The MS‐400 phased array transducer (30 MHz) was rotated at 90° to obtain a parasternal short‐axis view of the LV, and B‐mode recordings were obtained from a long‐axis view of the left ventricular. The left ventricle internal diameter in diastole (LVIDd) and systole (LVIDs), left ventricular ejection fraction (LVEF) and fractional shortening (FS) were then calculated by the echocardiographic system on M‐mode short video clip [27].

### 2.3 Assessment of Myocardial Injury, Collagen Content and Atrophy

The stored cardiac tissues were fixed in 4% paraformaldehyde, embedded in paraffin and sectioned at 5‐μm thickness. In order to assess the structure of the heart muscle, we utilised haematoxylin and eosin (H&E) staining. A pathologist was required to evaluate abnormalities on cardiac tissues and then slides were assigned to four classes, which are 0 (none, very little), +1 (mild), +2 (moderate), and +3 (severe). Masson staining was utilised to assess collagen content and the collagen volume fraction was quantified using Image‐Pro Plus 7.0 (Media Cybernetics, Rockville, USA) as previously described [28]. In addition, WGA (L4895, Sigma) staining was used to evaluate to the cross‐section size of the cardiomyocytes. The Leica DM3000 biological microscope (Germany) was utilised to capture the images.

### 2.4 Gut Microbiota 16 s RNA Sequencing and Untargeted Metabolomics

Serum samples and stools collected from all mice were stored at −80°C and analysed as previously described [16]. Detailed methods were shown in the Supporting Information.

### 2.5 RNA‐Sequencing

In accordance with the manufacturer's guidelines, total RNA was meticulously isolated and purified from heart tissue utilising TRIzol reagent (Invitrogen, Carlsbad, CA, USA). The RNA integrity was assessed by electrophoresis with denaturing agarose gel. Following this, the poly(A) RNA was fragmented into tiny segments, which were then reverse‐transcribed to craft cDNA, employing SuperScript II Reverse Transcriptase (Invitrogen). After a series of pretreatments, the ligated products were amplified with PCR. Finally, 2 × 150 bp paired‐end sequencing (PE150) was performed on an Illumina Novaseq 6000 (LC‐Bio Technology Co. Ltd., Hangzhou, China) in accordance with the vendor's recommendations. Subsequent bioinformatics analyses were performed using R (Version 4.3.0). Differential expression analyses were conducted using DESeq2 (R packages v1.38.1) with a cut‐off set at a 1.5‐fold change and a significance threshold of *p* < 0.05 [29].

### 2.6 Tissue Preparation, Single‐Nucleus RNA Sequencing (snRNA‐Seq) and Statistical Analysis

Mice from the control and DIC groups (*n* = 3) were sacrificed and isolation of nuclei from ventricle tissue were analysed, as previously described [30]. Gel bead‐in‐emulsions (GEMs) were generated by encapsulating single‐cell suspensions into droplets through the 10× Genomics Chromium instrument, with an aim to capture approximately 8000 single cells. This process adhered to the specific manufacturer's directives available for the 10× Genomics Chromium Single‐Cell 3′ kit (V3). Pursuant to this, cDNA amplification and library construction steps were carried out in line with the outlined standard protocol. The libraries were subsequently sequenced on the sophisticated Illumina NovaSeq 6000 platform (involving a paired‐end multiplexing run of 150 bp), facilitated by LC‐Bio Technology Co. Ltd., (Hangzhou, China). This was performed to a minimum sequencing depth of 20,000 reads for each cell, ensuring an optimal data set for downstream analyses. We utilised the CellRanger v.4.0 (10× Genomics) software to align sequencing data with the GRCm38 reference genome. Subsequently, the output from CellRanger was imported into the Seurat (v.5.1.0) software for unsupervised clustering analysis. During this process, we eliminated genes detected in less than three cells and ensured each cell expressed at least 50 genes. We also retained only cells that expressed more than 50 genes and had a mitochondrial gene expression rate of less than 5%. For data visualisation, we used the Seurat package to perform in‐depth analysis. Firstly, we used Seurat's LogNormalize function to normalise the expression values of the genes. Then, we ran the FindVariableGenes function and executed a principal component analysis on the normalised expression matrix of the highly variable genes identified. Following this, we used a method based on weighted shared nearest neighbour graphs, and conducted unsupervised cell segregation based on the first 10 principal components. In order to find genes unique to the clusters, we located the marker genes within Seurat through the FindAllMarkers function. Finally, genes within the target cluster that accounted for more than 25% of the total cell count and had a log fold change greater than 1 were selected as marker genes. Ultimately, we determined cell types based on previously validated markers. RunUMAP subsequently used to bring UMAP clustering into visible representation. Subsequently, enrichment analyses‐for Kyoto Encyclopedia of Genes and Genomes (KEGG) pathways‐were conducted [31].

### 2.7 Cell Treatment and Transfection

H9C2 cells were maintained in DMEM (Sigma) with 10% fetal bovine serum (FBS HyClone) at 37°C in an incubator with 5% CO_2_. MDA‐MB‐231 cells were maintained in RPMI Medium 1640 (Gibco) supplemented with 10% FBS. H9C2 cells were treated with DOX (1 μM) and ILA at a wide range of final concentrations (0.1–100 mM) for 48 h, and the optimal concentration of ILA were used for subsequent analysis. Cells were cultured with Fer‐1 (1 μM) for 24 h and harvested to investigate the role of ferroptosis in DOX‐induced cell injury. For gene manipulation, cells were transfected with aryl hydrocarbon receptor (AhR) siRNA (si‐AhR), the corresponding si‐NC, Nrf2 siRNA (si‐Nrf2), or si‐NC. The sequences are listed in Supplemental Table S2. Transfection was performed using Lipofectamine 3000 (Invitrogen) according to the manufacturer's instructions.

### 2.8 Statistical Analysis

Statistical analyses were performed using the GraphPad Prism 9 software (GraphPad Software Inc., San Diego, California, USA). Data are shown as mean ± SD. Differences between groups for normal data were analysed using one‐way analysis of variance, followed by Tukey's post hoc analysis. The Wilcoxon rank‐sum test was used to compare non‐normally distributed data. Statistical significance was set at *p* < 0.05. difference. Correlations between two parameters were calculated using the Spearman algorithm in R Version 4.2.3.

## 3 Results

### 3.1 ILA Is Decreased in DOX‐Treated Mice due to Imbalance of Gut Microbiota

**FIGURE 1:**
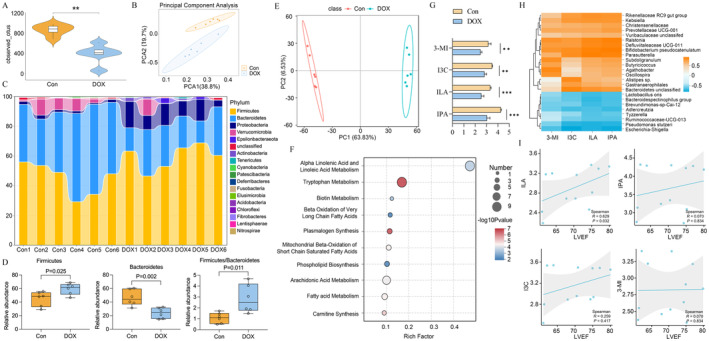
DOX treatment caused dysregulated serum microbial metabolites and reduced ILA levels by disturbing the balance of gut microbiota (*n* = 6). (A) Scatter plot of scores from principal component analysis (PCA) of serum metabolome. (B) Histogram of the most 10 Kyoto Encyclopedia of Genes and Genomes (KEGG) pathways with significant difference between the DOX and control groups. (C) The changes of tryptophan metabolites 3‐Methyldioxyindole (3‐MI), indole‐3‐carbinol (I3C), indole‐3‐lactic acid (ILA), indole‐3‐propionic acid (IPA), and in the serum of the mice from the control and DOX group. (D) The observed operational taxonomic units (OTUs) number of intestinal bacteria was examined by 16S‐rRNA high‐throughput sequencing. Significant differences are obtained by Wilcoxon rank‐sum test. (E) The β‐diversity of intestinal bacteria was compared by the PCA in mice treated with DOX. (F) The relative abundance of the gut bacterial phylum in mice treated with or without DOX. (G) The relative abundance of the Firmicutes, Bacteroidetes, and Firmicutes/Bacteroidetes (F/B) ratio in DOX‐treated mice. (H) Spearman correlation analysis showed the relationship between the altered four tryptophan metabolites and the altered microbiota. (I) Spearman correlation analysis showed the relationship between the altered four tryptophan metabolites and LVEF of mice. *p < 0.05, **p < 0.01, ***p  < 0.001.

Firstly, we measured bacterial α‐diversity presented by observed outs and assessed β‐diversity using principal component analysis (PCA). The results showed significant differences in both α‐diversity (Figure 1A) and β‐diversity (Figure 1B) between the mice in the Con and DOX group, suggesting the changes of gut microbiota upon DOX stimulating. At the phylum level, a notable interindividual variation was observed in the gut microbiota composition of mice subjected to DOX (Figure 1C). The balance between Firmicutes and Bacteroidetes (F/B) is often regarded as a health indicator, with a higher F/B ratio suggesting an imbalance in the microbiota [32]. Specifically, the abundance of Firmicutes was increased, Bacteroidetes was decreased and the F/B ratio was increased in the DOX group when comparable to the Con group (Figure 1D). As changes in gut microbiota generally mediate cardiovascular protective effects through their metabolites [33], we additionally analysed the alterations in serum metabolites in DOX‐treated mice. The results of PCA in Figure 1E revealed marked group differences between the control and DOX group. Using the criterion *p* ≤ 0.05 and |log2 (fold change) | ≥ 1.25, 1151 up‐regulated metabolites and 725 down‐regulated metabolites were identified. Subsequent KEGG enrichment analysis of changed metabolites showed alpha linolenic acid and linoleic acid metabolism, tryptophan metabolism, and biotin metabolism etc., made up the top 10 impacted pathways (Figure 1F). As tryptophan metabolism is closely linked with many progression of various inflammatory diseases, including cardiovascular diseases [34]. We therefore, focused on tryptophan metabolism, which is one of the most changed metabolic pathways (Figure S1). The levels of microbiota‐derived tryptophan metabolites were compared and we found DOX caused significantly downregulation of indole‐3‐lactic acid (ILA), indole‐3‐propionic acid (IPA), indole‐3‐carbinol (I3C) and 3‐Methyldioxyindole (3‐MI) (Figure 1G).

Previous studies have reported that ILA was secreted by Bifidobacterium [35]. Here, at family level, we validated that ILA, IPA, IAA and 3‐MI were all positively correlated with Parasutterella, Bifidobacterium_pseudolongum, Alistipes_sp., Ralstonia and Defluviitaleaceae_UCG‐011 (Figure 1H). Furthermore, we investigate the relationship between tryptophan metabolites and cardiac function. We found only ILA showed positively correlation with LVEF in mice (*R* = 0.629, *p* = 0.032) and other three tryptophan metabolites showed no significant correlation with LVEF (Figure 1I). Collectively, we identified that DOX may decrease ILA levels by disrupting the balance of gut microbiota.

### 3.2 Addition of ILA Protects DOX‐Induced Cardiac Impairment and Mitochondrial Injury

**FIGURE 2:**
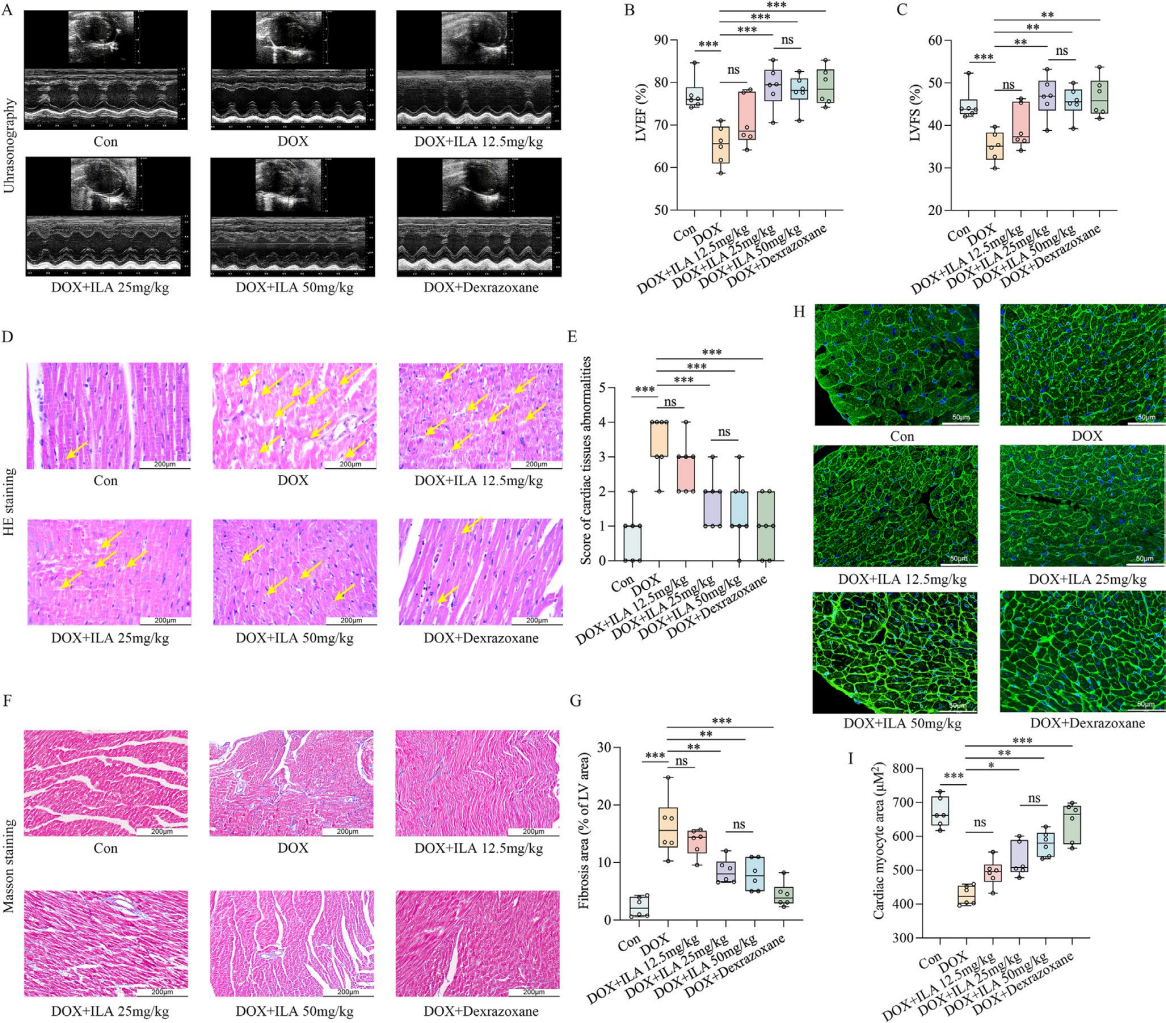
ILA exerts cardioprotective effects on DOX‐induced cardiotoxicity (*n* = 6). (A) Anatomic M‐mode echocardiography and corresponding electrocardiographic images of hearts from DOX‐treated mice, with or without dexrazoxane or different concentration of ILA (12.5, 25 and 50 mg/kg) treatment. Changes of echocardiographic (B) left ventricular ejection fraction (LVEF) and (C) left ventricular fraction shortening (LVFS) in the indicated groups. (D) Representative H&E staining images of the heart tissue section from mice treated with DOX and/or different concentration of ILA, and dexrazoxane. (E) Score of cardiac injury was classified as 0 (none, very little), +1 (mild), +2 (moderate), or +3 (severe). Bar = 100 μm. (F) Representative 200× images of myocardial tissue sections stained with Masson's trichrome. (G) Quantification of the fractional area of cardiac fibrosis based on 5 randomly selected fields of stained myocardial tissue sections. (H) Wheat germ agglutinin (WGA) staining of myocardial tissue sections to evaluate cardiac atrophy. (I) Quantitation of myocardial cell size of DIC mice in the indicated groups. The *p*‐value was calculated by one‐way ANOVA (Dunnett's or Sidak multiple comparisons tests). NS, no significance; *p < 0.05, **p < 0.01, ***p < 0.001.

We then analysed the functional role of ILA in DIC. The echocardiography results showed that the addition of a series of concentrations of ILA (12.5, 25, and 50 mg/kg) significantly rescued the DOX‐induced reduction in LVEF and LVFS (Figure 2A–C). Further, 25 and 50 mg/kg ILA showed cardioprotective effects equivalent to that of dexrazoxane, the only protective agent approved by the FDA for the treatment of DIC [36]. H&E staining was performed to evaluate the cardiomyocyte morphology. The DOX group exhibited significant myocardial damage, including karyolysis, muscle fibre disorganisation, and interstitial edema (indicated by arrows in Figure 2D), in contrast to the control group. However, treatment with 25 and 50 mg/kg ILA, as well as dexrazoxane, notably improved DOX‐induced cardiac injury (Figure 2D,E). We also investigated the role of ILA in DOX‐induced cardiac fibrosis and atrophy. The results of Masson staining and WGA assays demonstrated that DOX treatment resulted in the accumulation of collagen and cardiac atrophy, whereas the addition of 12.5, 25 and 50 mg/kg ILA significantly reduced collagen deposition (Figure 2F,G) and rescued the size of cardiomyocytes (Figure 2H,I). We found no statistically significant differences between the 25 and 50 mg/kg ILA groups. In addition, because mitochondrial damage is a key mechanism in DIC [37], we tested the role of ILA in DOX‐induced mitochondrial injury. The results of TEM revealed that DOX caused a loose mitochondrial structure, incomplete mitochondrial ridges, and some vacuoles, and this damage could be effectively alleviated by treatment with different concentrations of ILA and Dexrazoxane treatment (Figure S2A).

We analysed the effects of ILA on DOX‐induced cardiomyocyte death and investigated whether ILA affected the anticancer efficacy of DOX. The CCK‐8 assay was performed under identical conditions. ILA did not induce cytotoxicity in H9C2 cells and slightly promoted cell proliferation at the concentration of 0.1–10 mM, both at 24 and 48 h (Figure S3A,B). Viability of H9C2 cells decreased following DOX treatment, which was reversed by 0.1–10 mM of ILA (Figure S3C). ILA showed no cytotoxicity against MDA‐MB‐231 cells (Figure S3D), and 0.1, 1 and 10 mM ILA in combination with DOX significantly reduced the viability of MDA‐MB‐231 cells compared with that of the DOX treatment group (Figure S3E). Taken together, these results indicated that ILA did not affect the anticancer efficacy of DOX or alleviate DOX‐induced cell death. Subsequent analysis found that 0.1, 1, and 10 mM of ILA treatment suppressed DOX‐induced disruption of mitochondrial membrane potential (Figure S2B,C) and mtROS (Figure S2D–G). Taken together, these data indicate that ILA are protective against DIC.

### 3.3 ILA Alleviates DOX‐Induced Ferroptosis in Mice

**FIGURE 3:**
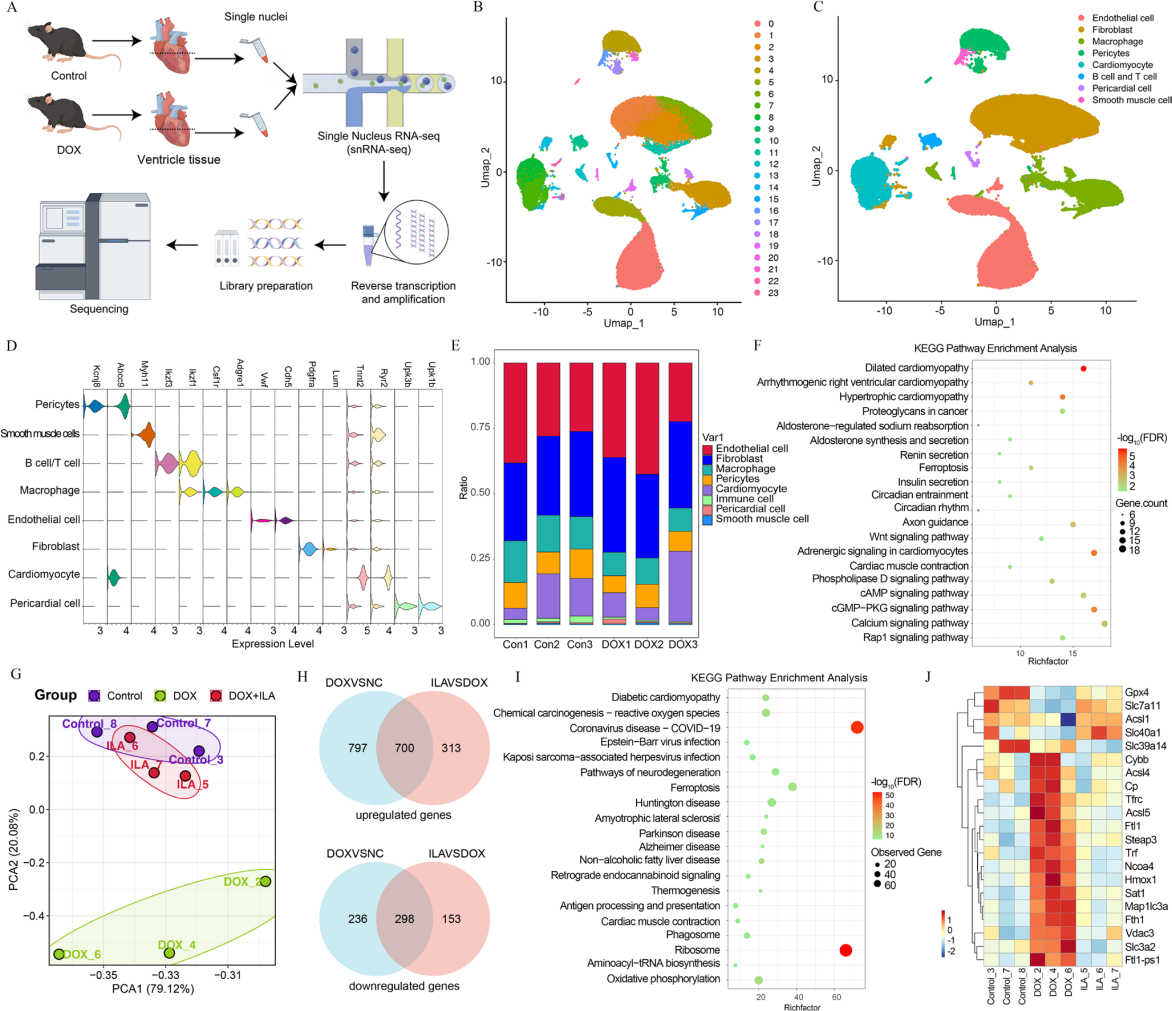
Ferroptosis is associated with DOX‐induced cardiotoxicity. (A) Schematic diagram showing the single nucleus RNA‐sequencing experimental design and analytical workflow (*n* = 3). (B) UMAP (uniform manifold approximation and projection) plot of all the single nuclei cells. (C) UMAP of integrated data identified eight cell lineages (resolution ratio = 0.5). (D) The classic marker genes in each cell lineage. (E) Bar plot showed the cell proportion among different groups. (F) Dot plots showing the top 20 biological terms for the differentially expressed genes processes of cardiomyocytes by KEGG pathway enrichment analysis. (G) Principal Component Analysis diagram of differentially expressed genes in the indicated groups. (H) Venn diagram of core co‐targeted upregulated and downregulated genes of ILA (indole‐3‐lactic acid) and doxorubicin. (I) KEGG pathway analysis of differentially expressed genes induced by doxorubicin (20 mg/kg) or ILA (25 mg/kg). (J) A heatmap showing the expression of ferroptosis‐related genes in ventricular tissue from mice treated with doxorubicin (20 mg/kg) or ILA (25 mg/kg) (*n* = 3).

To elucidate the mechanism of DOX‐induced myocardial damage and screen new drugs with few side effects to prevent DIC, we conducted single‐nucleus RNA sequencing (snRNA‐seq) on heart samples from control and DIC mice (*n* = 3, Figure 3A and Figure S4A). Using unbiased clustering analysis, we identified and visualised 23 clusters (Figure 3B and Figure S4B). Eight cell types from six hearts were eventually separated based on specific markers (Figure 3C). As shown in Figure 3D and Figure S4C, cells were identified by examining the expression of known lineage markers in cardiomyocytes (marked by *Tnnt2* and *Ryr2* [38]), fibroblasts (marked by *Pdgfra*, and *Lum* [39]), endothelial cells (marked by *Vwf* and *Cdh5* [39]), macrophage (marked by *Ikzf1*, *Csf1r*, and *Adgre1* [40]), smooth muscle cells (marked by *Myh11*), pericardial cell(marked by *Upk1b* and *Upk3b* [39]), pericytes (marked by *Kcnj8* and *Abcc9* [41]), and B cell/T cell (marked by *Ikzf3* and *Ikzf1* [42]). The percentage of cardiomyocyte nuclei in the ventricular myocardium was approximately 18% (Figure 3E). To further study the characteristics of cardiomyocytes after chemotherapy, we used differentially expressed genes (DEGs) in the cardiomyocytes to compare their transcriptional profiles and pathway enrichment between the two groups. After DOX treatment, hypertrophic cardiomyopathy, adrenergic signalling in cardiomyocytes, dilated cardiomyopathy, calcium signalling pathway and ferroptosis pathways were significantly enriched (Figure 3F).

To investigate the mechanisms through which ILA improves DIC, we further conducted bulk RNA‐seq on the DOX‐stimulated heart tissues. Principal component analysis (PCA) suggested good reproducibility of the samples within each group, with the DOX + ILA group and Control group being distinct from DOX group (Figure 3G). RNA‐seq analysis of control and DOX hearts identified 1498 upregulated and 534 downregulated DEGs (defined using log 2‐fold change > 1 and *p* value < 0.05). A total of 1498 upregulated and 534 downregulated DEGs were identified between the DOX + ILA group and DOX group (Figure S5A). The heatmap of these DEGs were shown in Figure S5B. The upregulated DEGs shared by the three groups had 700 genes, and downregulated DEGs shared by the three groups had 298 genes (Figure 3H). Subsequently, the total 998 DEGs were used for GO and KEGG enrichment analysis. The top five GO terms were mainly related to cytoplasm, mitochondrion, protein binding and ribosome (Figure S5C). Comparative KEGG pathway analysis revealed that these differentially expressed genes were significantly enriched for coronavirus disease, ribosome, oxidative phosphorylation, ferroptosis and glutathione biosynthesis (Figure 3I). Recently, a series of studies identified that ferroptosis a new therapeutic target against DIC [43, 44]. Consistent with these findings, our results also indicate the involvement of ferroptosis in DIC, prompting us to conduct further investigations into its mechanisms. By using cardiac transcriptome sequencing, we confirmed that after DOX treatment, ferroptosis‐related genes such as *Gpx4* (glutathione peroxidase 4), *Slc7a11* (solute carrier family 7a member 11) were significantly downregulated, while *Acsl4* (acyl‐coenzyme A synthetase long‐chain family member 4), *Ftl1* (ferritin light polypeptide 1) and *Ncoa4* (nuclear receptor coactivator 4) were upregulated; whereas, addition of 25 mg/kg ILA significantly reversed above changes (Figure 3J). Consequently, we hypothesized the involvement of ferroptosis in the protective effects of ILA against DIC.

### 3.4 ILA Improves DOX‐Induced Ferroptosis Depending on AhR

**FIGURE 4:**
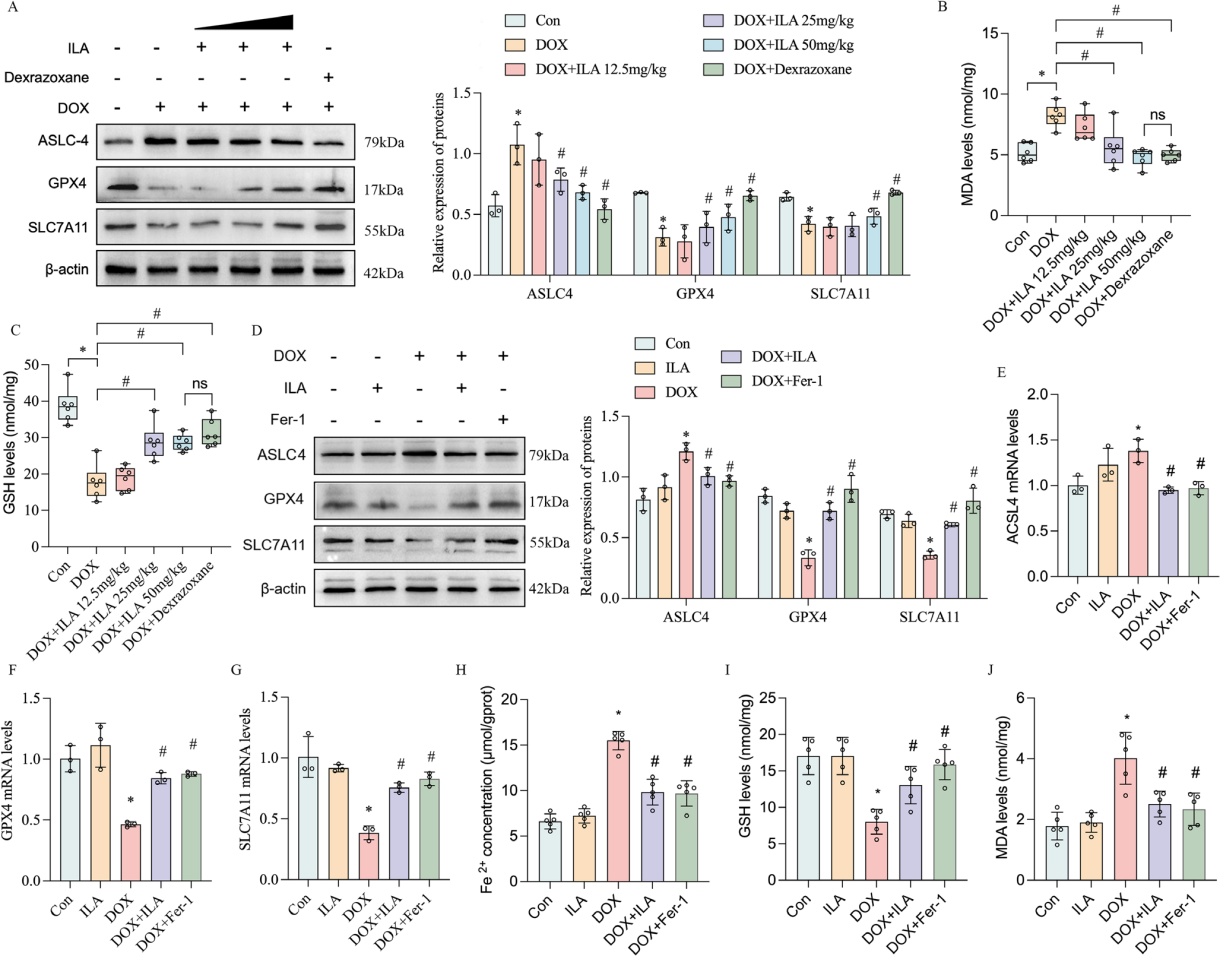
ILA attenuates DOX‐activated cardiomyocyte ferroptosis, both in vivo and in vitro. (A) Western blot was used to determine the changes of ferroptosis marker ACSL4, GPX4 and SLC7A11 in heart homogenates from mice treated with DOX and/or different concentration of ILA, and dexrazoxane. Relative expression of (B) malondialdehyde (MDA), (C) glutathione (GSH) levels in heart homogenates from mice treated with DOX and/or different concentration of ILA and dexrazoxane, were detected using commercial assay kits. (D) Protein expression of ACSL4, GPX4, and SLC7A11 were detected in H9C2 cells upon DOX, ILA and/or ferroptosis inhibitor ferrostatin‐1 (Fer‐1) treatment. (E–G) Relative expression of ACSL4, GPX4 and SLC7A11 mRNA in H9C2 cells. (H) The content of Fe^2+^ in H9C2 cells was assessed by the commercial kit. (I, J) Relative expression of MDA and GSH in H9C2 cells were determined after indicated treatments. The *p*‐value was calculated by one‐way ANOVA (Dunnett's or Sidak multiple comparisons tests). ns, no significance; *p < 0.05 versus Con; #*p* < 0.05 versus DOX.

As expected, treatment with ILA and dexrazoxane inhibited DOX‐induced ferroptosis, as evidenced by increased GPX4 and SLC7A11, and decreased ACSL4 protein levels (Figure 4A). In addition, DOX increased the levels of MDA (malondialdehyde, Figure 4B) and decreased the amount of GSH (glutathione, Figure 4C) in cardiac tissues, whereas treatment with 25 mg/kg and 50 mg/kg ILA and dexrazoxane partially reversed these changes. In DOX‐treated H9C2 cells, ILA remarkably reversed the decrease in GPX4 and SLC7A11 and increased ACSL4 caused by DOX, both at the protein and mRNA levels, similar to the effect of the ferroptotic inhibitor Ferrostatin‐1 (Fer‐1, Figure 4D–G). In addition, we found that stimulation with DOX notably increased iron and MDA levels and decreased GSH levels. Both ILA and Fer‐1 induction led to a decrease in iron and MDA levels and an increase in GSH levels in H9C2 cells (Figure 4H–J).

**FIGURE 5:**
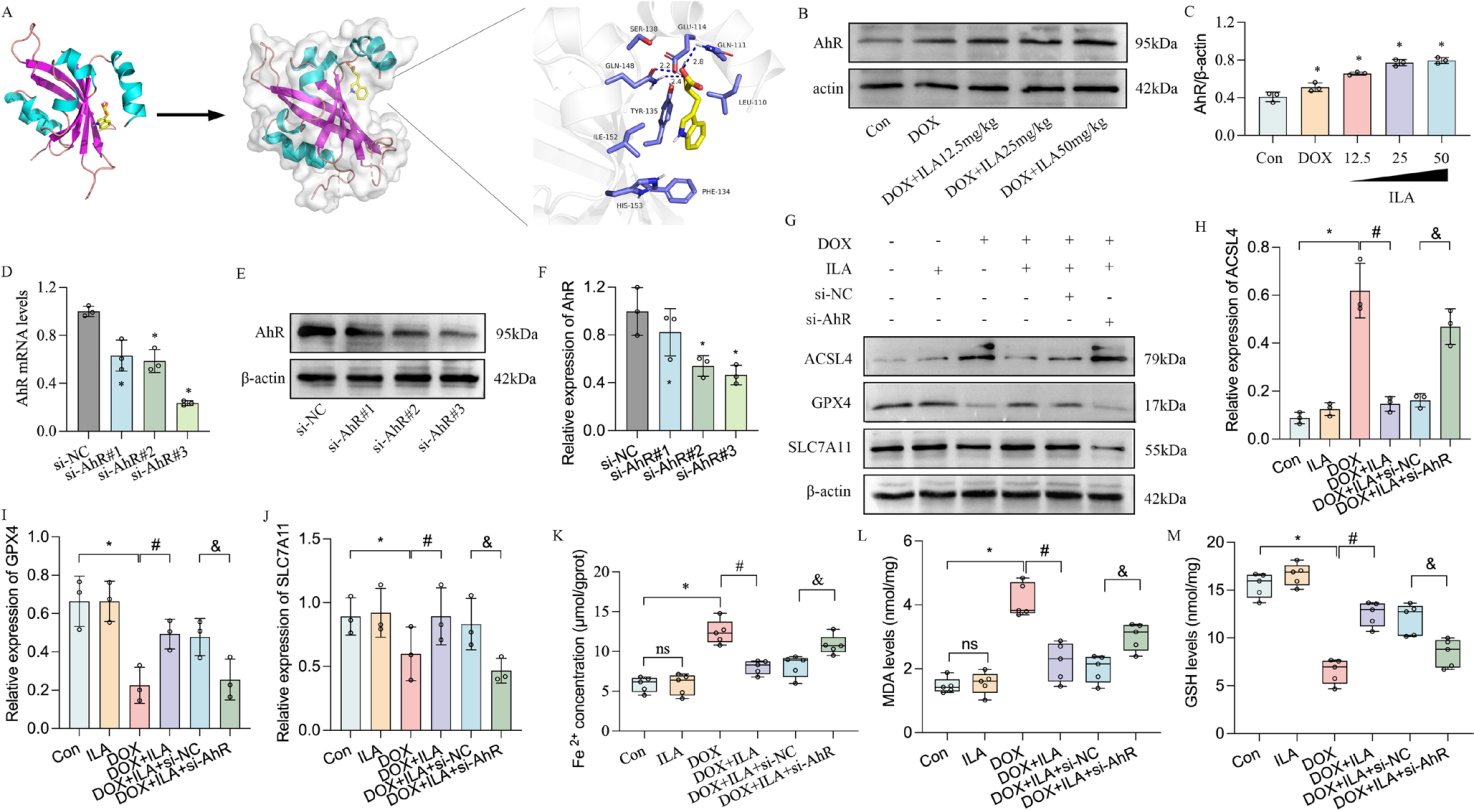
ILA inhibits DOX‐induced ferroptosis by upregulating aryl hydrocarbon receptor (AhR). (A) The binding mode and secondary structure of AhR were demonstrated by a cartoon model. (B, C) Protein level of AhR was assessed in heart homogenates from mice treated with DOX and/or different concentration of ILA. (D) Small interfering RNA against AhR was designed and after transfection with AhR siRNAs, its expression was determined by RT‐qPCR analysis. (E, F) The changes of protein level of AhR in H9C2 cells transfected with AhR siRNAs. (G–J) Protein expression of ACSL4, GPX4 and SLC7A11 were detected in H9C2 cells transfected with AhR siRNA, under DOX condition. (K) The content of Fe^2+^ in H9C2 cells was assessed by the commercial kit after inhibition of AhR. (L, M) Relative expression of MDA and GSH in H9C2 cells were determined after indicated treatments. The *p*‐value was calculated by one‐way ANOVA (Dunnett's or Sidak multiple comparisons tests). *p < 0.05 versus Con; #*p* < 0.05 versus DOX; &*p* < 0.05 versus DOX + ILA + si‐NC.

A previous study identified AhR as a target of ILA [45]. To assess the ability of ILA to bind to its target AhR, AutoDock Vina was used for molecular docking analysis. The results showed promising hydrogen bonding interactions between ILA and AhR, indicating a strong binding affinity between them (Figure 5A). We found that the protein expression of AhR was slightly increased by DOX and further increased with increasing concentrations of ILA (Figure 5B,C). This led us to hypothesize that ILA could potentially provide cardioprotective benefits by acting on AhR. An siRNA targeting AhR was designed to inhibit AhR expression in H9C2 cells, which resulted in decreased AhR mRNA and protein levels (Figure 5D–F). Western blotting revealed that ILA elevated the expression of GPX4 and SLC7A11 and decreased the expression of ACSL4 at the protein and mRNA levels. However, these effects were nullified by AhR knockdown (Figure 5G–J). Subsequent colorimetric determination showed that ILA treatment was capable of reversing the induction effects of iron and MDA as well as increasing GSH levels. However, these therapeutic effects were abolished in the absence of AhR (Figure 5K–M). Overall, our findings suggested that AhR plays a critical role in mediating the cardioprotective effects of ILA against DOX‐induced ferroptosis in cardiomyocytes.

### 3.5 ILA Activates the Nrf2/HO‐1 Signalling Pathway Through AhR

**FIGURE 6:**
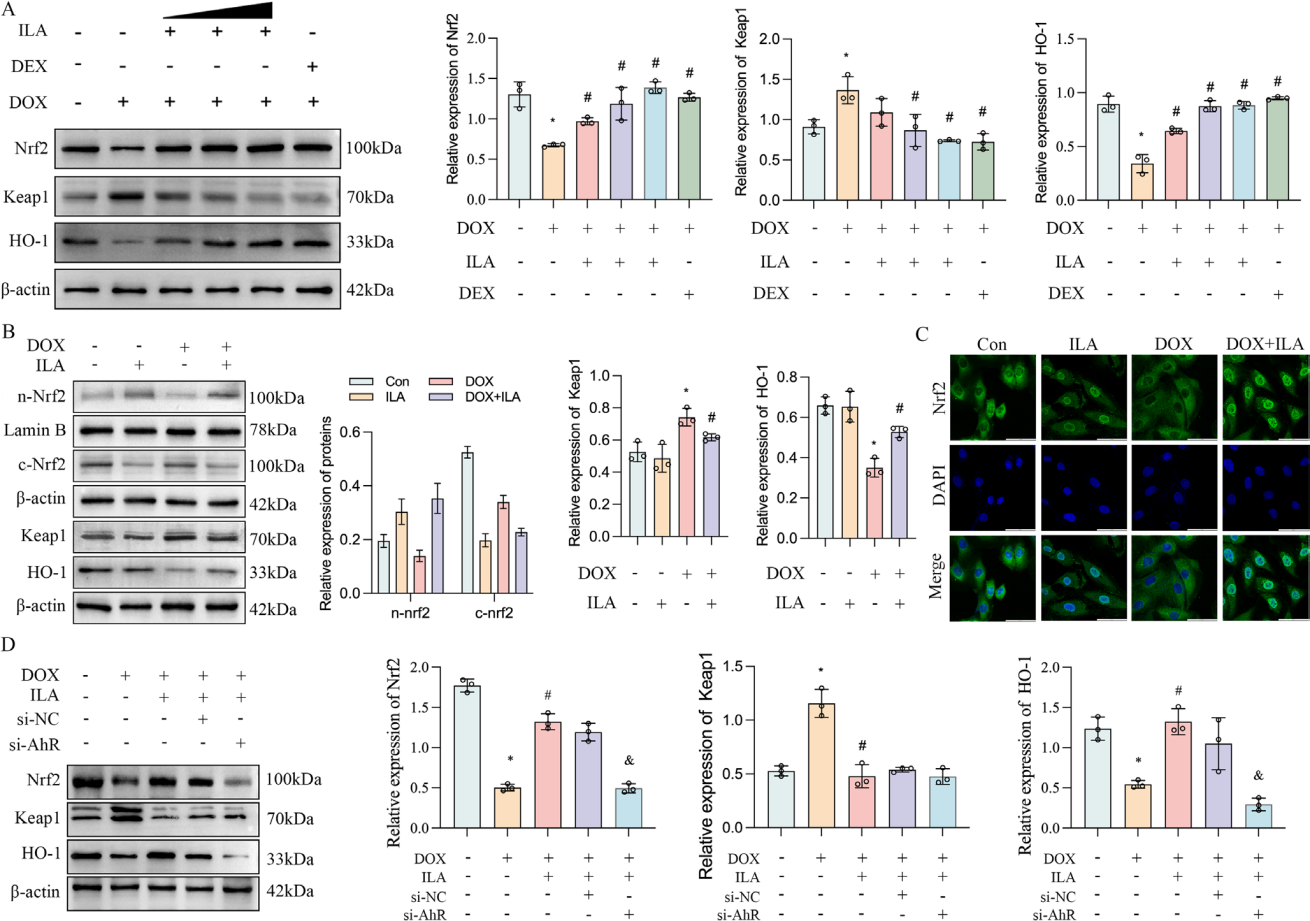
ILA activates the Nrf2/HO‐1 signalling pathway, in a AhR‐dependent way. (A) Western blot was used to determine the protein levels of Nrf2, HO‐1 and Keap1 in heart homogenates from mice treated with DOX and/or different concentration of ILA. (B) Western blot of nuclear and cytosol Nrf2 levels, HO‐1 and Keap1 in H9C2 cells in the indicated group. (C) Detection of the Nrf2 expression and translocation using immunofluorescence staining in H9C2 cells treated with DOX and/or ILA. (400×, Scale bar = 50 μm). (D) After inhibition of AhR, protein levels of Nrf2, HO‐1 and Keap1 in H9C2 cells were detected. The *p*‐value was calculated by one‐way ANOVA (Dunnett's or Sidak multiple comparisons tests). *p < 0.05 versus Con; #*p* < 0.05 versus DOX; &*p* < 0.05 versus DOX + ILA + si‐NC.

Pathway interaction prediction studies have suggested that endogenous ligands can activate AhR, which in turn activates downstream transcription factors, including NLE2L2, which encodes the Nrf2 protein by pathway interaction prediction (Figure S6). Hence, we examined whether the activation of the downstream pathway by AhR through endogenous ligands could trigger the Nrf2/HO‐1 signalling pathway in cardiac myocytes. Western blotting results showed that DOX decreased the levels of Nrf2 and HO‐1 and increased Keap1 protein, while the addition of ILA and dexrazoxane reversed the inhibitory effect of DOX on the Nrf2/HO‐1 signalling pathway in vivo (Figure 6A). Furthermore, we found that the expression of Nrf2 in the cell nucleus was increased by DOX treatment but reversed by ILA treatment. DOX decreased Nrf2 expression, whereas ILA supplementation increased the expression of Nrf2 in the cytosol of H9C2 cells (Figure 6B). Fluorescence confocal microscopy confirmed that ILA promoted Nrf2 nuclear translocation (Figure 6C). We then explored whether ILA activated the Nrf2 signalling pathway in an AhR‐dependent manner. As shown in Figure 6D, the decrease in Nrf2 levels induced by DOX was rescued by ILA. However, the effect of ILA on the low Nrf2 levels induced by DOX was no longer observed after AhR knockdown.

### 3.6 ILA Inhibits Ferroptosis via Activating the Nrf2/HO‐1 Signalling Pathway

**FIGURE 7:**
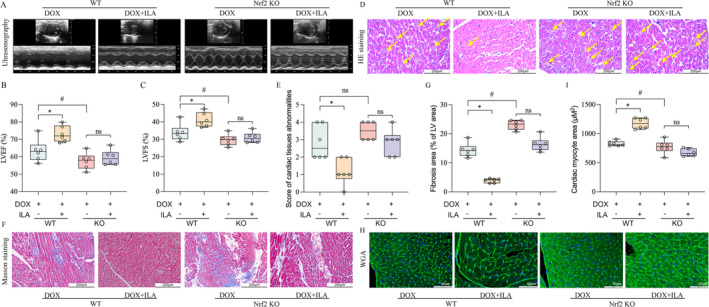
The inhibition of ILA for ferroptosis was abolished in Nrf2 KO mice (*n* = 6). (A) Anatomic M‐mode echocardiography and corresponding electrocardiographic images of hearts from Nrf2 knockout (KO) mice treated with DOX and/or ILA. Results of (B) LVEF, (C) LVFS in the indicated groups. (D) Representative H&E staining images of the heart tissue section in the indicated groups. (E) Score of cardiac injury was classified as 0 (none, very little), +1 (mild), +2 (moderate), or + 3 (severe). Bar = 100 μm. (F) Representative 200× images of myocardial tissue sections stained with Masson's trichrome. (G) Quantification of the fractional area of cardiac fibrosis based on 5 randomly selected fields of stained myocardial tissue sections. (H) Wheat germ agglutinin (WGA) staining of myocardial tissue sections to evaluate cardiac atrophy. (I) Quantitation of myocardial cell size in DIC mice in Nrf2 KO mice (*n* = 6). The *p*‐value was calculated by one‐way ANOVA (Dunnett's or Sidak multiple comparisons tests). ns, no significance; *p < 0.05 versus WT DOX ; #*p* < 0.05 versus KO DOX.

**FIGURE 8:**
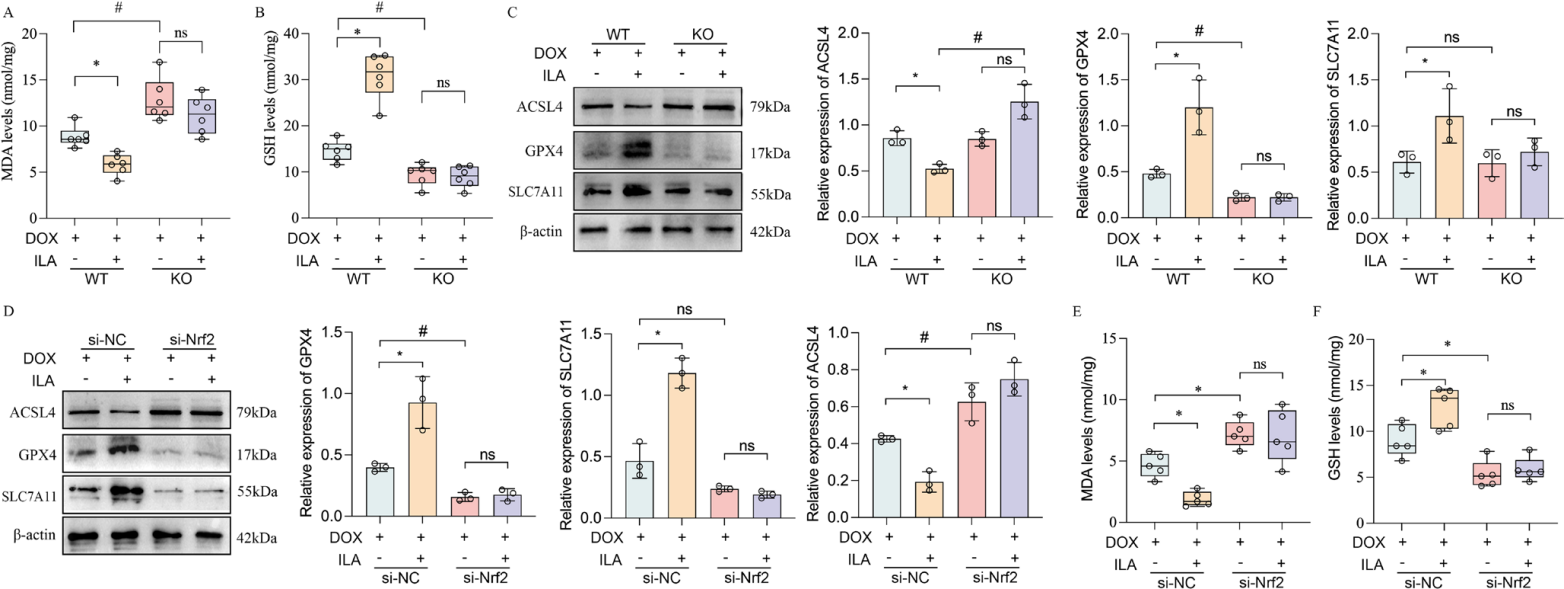
ILA inhibits DOX‐induced ferroptosis via activating the Nrf2/HO‐1 signalling pathway. Relative expression of (A) malondialdehyde (MDA), (B) glutathione (GSH) levels in Nrf2‐KO mice treated with DOX and/or 25 mg/kg ILA, were detected using commercial assay kits (*n* = 6). (C) Western blot was performed to measure the changes of ferroptosis marker ACSL4, GPX4 and SLC7A11 in heart homogenates from Nrf2‐KO mice treated with DOX and/or 25 mg/kg ILA (*n* = 3). (D) After inhibition of Nrf2 by using small interfering RNA (si‐Nrf2), protein expression of ACSL4, GPX4 and SLC7A11 were detected in H9C2 cells. (E, F) Relative expression of MDA and GSH in H9C2 cells transfected with si‐Nrf2 (*n* = 3). The *p*‐value was calculated by one‐way ANOVA (Dunnett's or Sidak multiple comparisons tests). ns, no significance; *p < 0.05 versus DOX + si‐NC; #*p* < 0.05 versus DOX + si‐Nrf2.

To confirm that Nrf2 is crucial for the protective effect of ILA against ferroptosis, we used Nrf2‐knockout mice to establish a DIC model. It was observed that the deletion of Nrf2 exacerbated DIC induced by DOX, as evidenced by decreased cardiac function. However, the protection of ILA for DIC was abolished in Nrf2^−/−^ mice (Figure 7A–C). Consistently, we observed lower myocyte disruption (Figure 7D,E), excessive collagen deposition (Figure 7F,G), and larger cardiomyocyte size (Figure 7H,I) in the hearts of Nrf2^−/−^ mice compared with those in the WT mice upon DOX condition. However, compared to the mice in the WT group, addition of ILA had non‐significant alleviation on cardiac function (Figure 7A–C), histological injury (Figure 7D,E), fibrosis (Figure 7F,G), and atrophy (Figure 7H,I) in Nrf2^−/−^ mice. Moreover, compared to WT group, the MDA levels were increased, and GSH levels were decreased further in Nrf2^−/−^ mice. Deletion of Nrf2 invalidated the anti‐lipid peroxidation function of ILA (Figure 8A,B). In Nrf2 deficiency mice, DOX caused more obvious mitochondrial structural change than those in WT mice (Figure S8A), and consistently, the inhibition of ILA for ferroptosis was also eliminated in Nrf2^−/−^ mice (Figure 8C). In vitro, the Nrf2 expression in H9C2 cells was silenced through the transfection with Nrf2 siRNA (Figure S7). The effects of ILA on DOX‐induced mitochondrial membrane potential and mtROS levels were abrogated after the inhibition of Nrf2 in H9C2 cells (Figure S8B). In vivo, a decrease in the levels of GPX4 and an increase in ACSL4 levels were observed after the silencing of Nrf2 in H9C2 cells. In agreement with the in vivo results, we observed an increase in the protein levels of GPX4 and SLC7A11 and a decrease in ACSL4 protein by ILA treatment. However, these levels were not reversed by ILA treatment after Nrf2 knockdown (Figure 8D). In addition, Nrf2 knockdown increased the levels of MDA and decreased GSH levels in DOX‐treated cells, and the anti‐lipid peroxidation ability of ILA was abolished (Figure 8E,F). In summary, we found that ILA inhibited ferroptosis in cardiomyocytes by activating the AhR‐mediated Nrf2/HO‐1/GPX4 signalling pathway and protected against DIC.

## 4 Discussion

The metabolic products generated by the microbiome play a pivotal role in connecting the gut microbiota to host physiology. Verification of the functions of microbial metabolites may assist in the development of new therapeutic approaches for cardiovascular diseases [46, 47]. Previous studies have suggested that microbial metabolites have beneficial effects in experimental liver [48], chronic kidney [49] and cardiovascular diseases [50]; however, the role of microbial metabolism in DIC remains unclear. By using multi‐omics analysis, this study uncovered a novel finding that DOX significantly decreased the expression of the tryptophan metabolite ILA by causing an imbalance in the gut microbiota, especially by lowering the abundance of Bifidobacterium. Our integrated experiments indicated that the administration of ILA effectively alleviated DIC by activating the AhR‐mediated Nrf2/HO‐1 signalling pathway.

Mice that received DOX displayed a compositional imbalance in the gut microbiota, different clusters of cellular processes, and metabolism [13]. Microbiota‐derived metabolites can activate several downstream signalling pathways via G protein‐coupled receptors or through direct immune cell activation [20] Tryptophan metabolites generated by the gut microbiota contribute to numerous physiological responses, engaging in both pro‐ and anti‐inflammatory effects via diverse cellular physiological mechanisms facilitated by AhR modulation [51]. Administration of ILA has been shown to be beneficial for anti‐inflammatory and eubiotic maintenance in liver disease via AhR‐mediated activation of intestinal immune cells [52]. AhR has the ability to bind a variety of foreign substances, such as flavonoids, polyphenolics, and indoles found in plants [53]. AhR is activated by several endogenous indole derivatives, such as indole‐3‐propionic acid [54], kynurenine [55] and ILA [56]. ILA can ameliorate colitis in caesarean‐born offspring by activating the AhR [57]. ILA have also been found to improve intestinal barrier damage by activating the AhR and Nrf2 signalling pathways [58]. In addition to their anti‐inflammatory effects, AhR agonists suppress ferroptosis by scavenging ROS and activating an AhR‐independent cell‐protective pathway [59]. Iron homeostasis is essential for cardiac function, and iron overload is a risk factor for DIC. Conversely, lower iron levels are associated with a lower risk of DIC [60], suggesting that cardiac iron regulation might be a potential treatment strategy. Dexrazoxane can reduce iron‐dependent DOX‐based oxidative stress [61] and is the only medication approved by the FDA for DIC, which suppresses ferroptosis in mice with cardiac injury [62]. However, studies on patients receiving chemotherapy have shown that dexrazoxane may reduce the sensitivity of cancer cells to chemotherapy and exacerbate myelosuppression, leading to the restrictive clinical use of dexrazoxane [63]. To the best of our knowledge, this is the first study to demonstrate the protective role of the tryptophan microbial metabolite ILA in alleviating cardiac ferroptosis in DIC. ILA showed efficacy equivalent to that of dexrazoxane in DIC mice and enhanced the antitumor activity of DOX in vitro, indicating its potential as a clinical agent for relieving DIC.

Mitochondria‐dependent ferroptosis plays a pivotal role in DIC development [5], In this study, the snRNA‐seq results revealed that DOX induces ferroptosis in cardiomyocytes. Subsequent bulk RNA‐seq identified that ILA treatment reversed the changes of ferroptosis‐related genes caused by DOX, suggesting ferroptosis is a valuable and promising strategy for preventing DIC. As expected, ILA treatment alleviated DOX‐mediated cell injury and activated AhR to inhibit myocardial ferroptosis, both in vivo and in vitro. Upon ligand binding, chaperones release AhR from its inactive state, allowing it to relocate to the nucleus [64]. The tryptophan metabolite, trans‐3‐indoleacrylic acid (IDA), acts as an endogenous ligand of AhR to upregulate ALDH1A3 expression and suppress ferroptosis [65]. In addition, AhR directly binds to the Nrf2 promoter, and kynurenine significantly enhances this binding capacity [66]. Furthermore, Zhang et al. demonstrated the existence of a crosstalk between AhR and Nrf2 in insects. They discovered that these transcription factors jointly regulate the expression of P450s, GSTs and UGTs, which are involved in xenobiotic metabolism and hormone pathway synthesis [67]. However, no studies have focused on the expression of AhR and its crosstalk with Nrf2 in DIC. Our previous studies demonstrated that the activation of the Nrf2 signalling pathway can prevent DIC [9, 17]. Hence, using bioinformatic analysis, we focused on the regulatory role of AhR in Nrf2 expression in DIC. In this study, through integrated experiments, we found that ILA upregulates AhR to promote *NFE2L2* transcription and activates the Nrf2/HO‐1 signalling pathway. Nrf2 is widely recognised as a key regulator of the body's antioxidant response as it directly regulates multiple downstream genes and enzymes responsible for maintaining the correct balance of reactive oxygen species within cells [68]. The Nrf2/heme oxygenase‐1 (HO‐1) signalling pathway is involved in DOX‐induced ferroptosis in vivo and in vitro [69]. A recent study found that ILA can suppress intestinal ischemia/reperfusion injury through the positive regulation of Nrf2 [23]. Our study demonstrated that ILA failed to protect DOX‐induced ferroptosis in Nrf2^−/−^ mice, highlighting the protective role of Nrf2 in DIC. Taken together, these results indicate that ILA exerts its cardioprotective effects through the AhR‐mediated Nrf2/HO‐1 signalling pathway.

This study has certain limitations. First, we performed the experiments only on DOX‐treated mice and H9C2 cells. Therefore, integrated microbiome‐metabolome analysis of patients with breast cancer treated with DOX should be performed. Currently, early and effective indicators for diagnosing DIC are lacking [70]. Further studies are required to explore intestinal metabolites as protective factors for DIC to improve the prognosis and quality of life in cancer patients. A recent study by Nemet et al. identified that plasma tryptophan metabolites (including indole‐3‐pyruvic acid, ILA, and indole‐3‐acetyl‐glutamine) are associated with incident (3‐year) major adverse cardiovascular events and poorer survival risks [71]. This finding contradicts most basic research [72] and additional studies are required to reveal the exact causal relationship between tryptophan metabolites and cardiovascular disease. In addition, knockdown of AhR only partially reversed the cardioprotective effects of ILA on DIC, indicating that ILA may exert its effect through another AhR‐independent mechanism. This encouraged us to further explore the exact mechanisms of ILA in cell ferroptosis. Furthermore, AhR contains many microbial ligands [73] and we investigated only the role of ILA in DIC. Therefore, other endogenous ligands of AhR such as indole‐3‐propionic acid, indole‐3‐acetic acid, and indole‐3‐aldehyde should be explored in future studies.

## 5 Conclusion

Collectively, our data show that the microbiota‐derived tryptophan metabolite ILA has protective effects against DOX‐induced myocardial ferroptosis by activating the Nrf2/HO‐1 signalling pathway in an AhR‐dependent manner. The current results provide motivation for considering nutritional interventions during the treatment of patients receiving DOX chemotherapy.

## Author Contributions

**Jiangfang Lian:** conceptualization (equal), data curation (equal), formal analysis (equal), funding acquisition (equal), project administration (equal), resources (equal), supervision (equal), visualization (equal), writing – original draft (equal), writing – review and editing (equal). **Hui Lin:** conceptualization (equal), data curation (equal), funding acquisition (equal), methodology (equal), resources (equal), software (equal), validation (equal), visualization (equal), writing – original draft (equal), writing – review and editing (equal). **Zuoquan Zhong:** conceptualization (equal), formal analysis (equal), methodology (equal), resources (equal), software (equal), validation (equal). **Yongfei Song:** data curation (equal), formal analysis (equal), methodology (equal), resources (equal), supervision (equal), visualization (equal), writing – original draft (equal). **Xian Shao:** conceptualization (equal), formal analysis (equal), methodology (equal), resources (equal), software (equal), validation (equal). **Jiedong Zhou:** conceptualization (equal), data curation (equal), formal analysis (equal), methodology (equal), project administration (equal), software (equal), visualization (equal). **Lili Xu:** conceptualization (equal), formal analysis (equal), investigation (equal), project administration (equal), software (equal), visualization (equal). **Zhenzhu Sun:** data curation (equal), methodology (equal), resources (equal), software (equal), visualization (equal), writing – original draft (equal), writing – review and editing (equal). **Yongyi Yang:** data curation (equal), investigation (equal), methodology (equal), resources (equal), software (equal), validation (equal). **Jufang Chi:** conceptualization (equal), funding acquisition (equal), investigation (equal), methodology (equal), project administration (equal), resources (equal), supervision (equal), visualization (equal), writing – original draft (equal). **Ping Wang:** data curation (equal), formal analysis (equal), methodology (equal), resources (equal), validation (equal), visualization (equal), writing – review and editing (equal). **Liping Meng:** conceptualization (lead), funding acquisition (lead), investigation (equal), supervision (equal), visualization (equal), writing – original draft (equal), writing – review and editing (equal).

## Ethics Statement

The studies involving human participants were reviewed and approved by the Animal Care and Use Committee of Shaoxing People's Hospital (No. 2020‐002).

## Conflicts of Interest

The authors declare no conflicts of interest.

## Supporting information


Supporting Information

## Data Availability

All data generated or analysed during this study are included in this published article. Additional datasets used during this study are available from the corresponding author on reasonable request.
