# Supplementary material for: Indole‐3‐Lactic Acid Inhibits Doxorubicin‐Induced Ferroptosis Through Activating Aryl Hydrocarbon Receptor/Nrf2 Signalling Pathway
Source: J Cell Mol Med. 2025 Jan 23;29(2):e70358. doi: 10.1111/jcmm.70358 (PMC11756996; doi:10.1111/jcmm.70358)
Supplement: Supplementary file 1 — Supporting Information [file JCMM-29-e70358-s001.docx]

**Supplementary Data**

**Title page**

**Indole-3-lactic acid inhibits doxorubicin-induced ferroptosis through activating aryl hydrocarbon receptor/Nrf2 signaling pathway**

**Authors**

Jiangfang Lian^1#^, Hui Lin^1#^, Zuoquan Zhong^2#^, Yongfei Song^1^, Xian Shao^2^, Jiedong Zhou^3^, Lili Xu^2^, Zhenzhu Sun^4^, Yongyi Yang^5^, Jufang Chi^6†^, Ping Wang^7†^, Liping Meng^7†^

**Institutions**

^# †^These authors share first authorship.

1. Department of Cardiology, the Affiliated Lihuili Hospital of Ningbo University Health Science Center, Ningbo, Zhejiang, 315211, China.

2. Central Laboratory of Medicine, Shaoxing People's Hospital, Shaoxing, 312000, China.

3. College of Medicine, Shaoxing University, Shaoxing, 312000, China.

4. Department of Cardiology, Enze Medical Research Center, Taizhou Hospital Affiliated to Wenzhou Medical University, Linhai, 317000, China.

5. Department of Gynaecology, The First Affiliated Hospital, College of Medicine, Zhejiang University, Hangzhou, 310020, China.

6. Department of Cardiology, Zhuji People's Hospital of Zhejiang Province, Zhuji Affiliated Hospital of Wenzhou Medical University, Zhuji, 311800, China.

7. Department of Cardiology, Shaoxing People’s Hospital (Shaoxing Hospital, Zhejiang University School of Medicine), Shaoxing, 312000, China.

**Supplementary Methods**

**Biochemical Analysis**

To evaluate the changes of lipid peroxidation, homogenate supernatant of myocardial tissues (9 mL 0.9% physiological saline and 1 g tissue) was collected after centrifugation at 12,000 rpm for 10 min, at 4 ℃. Treated H9C2 cells were collected to make the suspension cell followed by ultrasonic lysis. The levels of malondialdehyde (MDA, A003-1-2, Nanjing Jiancheng, China) and glutathione (GSH, A006-1-1, Nanjing Jiancheng, China) were enzymatically measured using a SpectraMax Plus Absorbance microplate reader (Molecular devices, CA). The intracellular ferrous iron (Fe^2+^) levels were determined according to the instructions provided with the iron assay kit (Cat.MAK025, Millipore Sigma).

**Transmission electron microscope**

A 0.5 cm^3^ sample of mice heart was taken, fixed for 2 hours in 2.5% glutaraldehyde in phosphate buffer, washed with a 1 mmol/L phosphoric acid solution, then fixed for 4 h in 1% osmium tetroxide. The tissues were precisely sliced into 1 μm thick portions, then 3% uranylacetate and lead citrate were applied to stain the slices, which were then examined using a transmission electron microscope (Titan G2 60‐300, FEI, Hillsboro).

**Cell proliferation assay**

Cell viability was quantified using the CCK-8 assay (MedChemExpress). H9C2 cells were plated at a density of 5,000 cells per well in 96-well plates. ILA was added at concentrations from 0.1 μM to 100 mM, and 10 μL of CCK-8 solution was introduced into each well at 48 h, and incubated for another 2 h to measure absorbance at 450 nm on the microplate reader. Cell viability was presented as the ratio of the absorbance of the treatment group to that of the control group.

**Detection of MtROS**

For detection of mtROS production, H9C2 cells were seeded at 70% confluency on a 6-well plate, and pre-stained 15 min prior to imaging with DAPI. The growth medium was then replaced with 0.5% FBS. At the start of imaging, MitoROS™ 580 working solution (AAT Bioquest, Pleasanton, CA, USA) for 10-30 min at 37 °C. Imaging was performed at 20x objective (Leica STELLARIS 5 confocal microscope). In addition, mtROS production was measured the fluorescence signal on a flow cytometry (Beckman Coulter CytoFLEX) at 485 nm/538 nm for DCFH-DA (Beyotime).

**Measurement of mitochondrial membrane potential**

A JC-1 kit (Beyotime) was sued to measure the mitochondrial membrane potential of the H9C2 cells. Briefly, approximately 1×10^5^ cells were mixed with 1 ml of JC-1 dye working solution and incubated for 20 min at 37°C. After washing twice with JC-1 dyeing buffer, the sample was visored under a Nikon Eclipse Ti‐U fluorescence microscope (Minato‐ku, Tokyo, Japan). The red/green fluorescence ratio was used to represent the mitochondrial membrane potential level.

**16S rRNA sequencing and taxonomic analysis**

Bacterial DNA from mice’s stools was extracted using the E.Z.N.A. ®Stool DNA Kit (D4015, Omega, Inc., USA). The total DNA was eluted in 50 μL of Elution buffer and stored at -80 °C until measurement in the PCR by LC-Bio Technology Co., Ltd, Hang Zhou, Zhejiang Province, China. PCR were performed to prepare amplicons using V3-V4 oligonucleotides (343F: 5’-TACGGRAGGCAGCAG-3’; 798R: 5’-AGGGTATCTAATCCT-3’). Samples were sequenced on an Illumina NovaSeq platform (Illumina, San Diego, CA, USA) according to the manufacturer’s recommendations. Paired-end reads were assigned to samples based on their unique barcode and truncated by trimming the barcode and primer sequence. Paired-end reads were merged using FLASH. Quality filtering of raw reads was performed under specific filtering conditions to obtain high-quality clean tags according to fqtrim (v0.94). Chimeric sequences were filtered using the Vsearch software (v2.3.4). After dereplication using DADA2, we obtained a feature table and feature sequence.

QIIME package was employed to select representative read. The α-diversity based on Chao1 and Shannon indexes were used to evaluate microbial diversity. β-diversity indicator principal co-ordinates analysis (PCoA) based on weighted Unifrac distance was conducted to assess microbial community alternations. The weighted Unifrac metric is weighted by the difference in the abundance of OTUs from each community. And linear discriminant analysis (LDA) effect size (LefSe) coupled with LDA was used to identify characteristic taxa among groups. Rings from the inside out represents phylum to genus levels, and sizes of circles indicate relative abundance of the taxon, and the graphs were plotted using the R package (v4.2.3).

**Ultra-performance liquid chromatography coupled with electrospray ionization and tandem mass spectrometry (UPLC-ESI-MS/MS) analysis for serum untargeted metabolites**

The collected serum samples were thawed on ice for 30 min., and metabolites were extracted with 50% methanol buffer. Briefly, 20 μL of the sample was extracted using 120 μL of precooled 50% methanol, vortexed for 1 min, and incubated at room temperature for 10 min. After centrifugation at 4,000 × g for 20 min, the supernatants were transferred into new 96-well plates and stored at -80 °C prior to LC-mass spectrometry (MS) analysis. All samples were acquired using the LC-MS system following machine orders. A high-resolution tandem mass spectrometer TripleTOF5600plus (SCIEX, Mereside, UK) was used to detect metabolites eluted from the column. Quadrupole time-of-flight MS was operated in both positive and negative ion modes. Pretreatments of the acquired MS data, including peak picking, peak grouping, retention time correction, second peak grouping, and annotation of isotopes and adducts, were performed using the XCMS software. CAMERA and metaX toolbox were implemented using the R software. Each ion was identified by combining the retention time and m/z data. Peak intensities were recorded, and a three-dimensional matrix containing arbitrarily assigned peak indices, sample names, and ion intensity information was generated. The online Kyoto Encyclopedia of Genes and Genomes (KEGG) database was used to annotate the metabolites by matching the exact molecular mass data (m/z) of samples with those from the database. Principal component analysis (PCA) was performed to detect outliers and evaluate batch effects using the pre-processed dataset. Student’s t-tests were conducted to detect differences in metabolite concentrations between the two phenotypes. The q value was adjusted for multiple tests using a false discovery rate (FDR; Benjamini–Hochberg). Supervised partial least squares-determinant analysis was conducted using metaX to compare different variables between groups.

**Quantitative real-time PCR (RT-qPCR)**

Total RNA was extracted from H9C2 cells using the TRIzolTM total RNA Extraction Kit (Takara, Otsu, Japan), and reverse transcription was performed using Prime Script RT reagent kit (Takara). AhR, GPX4, SLC7A11, Nrf2, HO-1 and Keap1 mRNA levels were detected using quantitative real-time reverse transcriptase PCR analysis using SYBR Premix Ex Taq kit (Takara). The reaction conditions being set as follows: (1) 95 °C for 10 min, (2) 40 cycles of 95 °C for 10 s and 60 °C for 30 s, (3) 72 °C for 15 s. GAPDH was used to normalize the target genes via the 2^−ΔΔCt^ method. The primers used in this study were shown in supplementary Table1.

**Western blot analysis**

Total protein was extracted from myocardial tissues or H9C2 cells using RIPA lysis buffer (Beyotime). Equal amounts (15 μg) of protein were subjected to electrophoresis and transferred to PVDF membranes (Millipore, MA). The membranes were incubated with the primary antibodies against AhR (#AF6278, 1:1000), GPX4 (DF6701, 1:1000), SLC7A11 (DF12509, 1:1000), β‐actin (AF7018, 1:5000, all obtained from Affinity Biosciences, Jiangsu, China), Nrf2 (ab62352), HO-1 (ab68477), Keap1 (ab227828) and LaminB (ab16048, all 1:1000. obtained from Abcam, Cambridge, MA) overnight at 4°C. The next day, the protein bands were treated with a Goat Anti-Rabbit IgG (H+L) HRP antibody (S0001, 1:5000; Affinity Biosciences), and visualized using an ECL detection kit (Beyotime) on a Tanon 5200Muti Multifunctional imaging system (Tanon Science and Technology).

**Immunofluorescence staining**

After fixation of H9C2 cells with a 4% formalin solution and treatment of 0.1% Triton X-100 in PBS, PBS was used to wash cells three times. The cells were then blocked using 1% BSA and incubated with Nrf2 overnight at 4 °C. The cells were then incubated with the secondary antibodies Goat Anti-Rabbit IgG H&L (Alexa Fluor 488, 1:1000) for 1 h. After DAPI staining, cells were observed under a fluorescence microscopy.

**Supplementary figures/tables**

**Supplementary Table 1. Primer pairs for qPCR analysis**

| Gene | Forward (5’-3’) | Reverse (3’-5’) |
| --- | --- | --- |
| *AhR* | GAGCACAAATCAGAGACTGG | TGGAGGAAGCATAGAAGACC |
| *Nrf2* | TTCACTAAACCCAAGTCCCAGCAT | AAGCCAAGCAGTGTGTCTCCATA |
| *HO-1* | GACTGCGAGGAGGACCAAAA | CAGCCCGTATATCTTGCCGT |
| *Keap1* | GCCGAGTCTGTTCTGGCTTATT | CATGGCAGCGTAAGTGTAAGCA |
| *GPX4* | CCTGGTCTGGCAGGCAC | GCTAGAGATAGCACGGCAGG |
| *SLC7A11* | AAATACGGAGCCTTCCACGAG | AGGAAAATCTGGATCCGGGC |
| *GAPDH* | AGGTCGGTGTGAACGGATTTG | TGTAGACCATGTAGTTGAGGTCA |

**Supplementary Table 2. Primer pairs for RNA interference**

| Genes |  | 5’-3’ primer sequence |
| --- | --- | --- |
| *si-AhR#1* | sense | TCAGCCCACCGTAACAAT |
|  | anti-sense | CAAACTTCTCGGCGTCAT |
| *si-AhR#1* | sense | AAATGACAAAGCCCTGATGG |
|  | anti-sense | GAACCCATCCTCGAAGTTCA |
| *si-AhR#1* | sense | TCGGGTGGTTTCCATTCAGACAGA |
|  | anti-sense | GGGTCTTCCATTTCAGGGTCCAAA |
| *si-NC* | sense | GTGTTGCCCCTGAAGAGCAT |
|  | anti-sense | GCTGGGACATTGAAAGTCTCA |
| *si-Nrf2* | sense | GACATGGATTTGATTGACATCCTTT |
|  | anti-sense | AAAGGAUGUCAAUCAAAUCCAUGUC |
| *si-NC* | sense | GACGGTATTAGTTAGACCTCTATTT |
|  | anti-sense | AAAUAGAGGUCUAACUAAUACCGUC |

**Supplementary Figures**


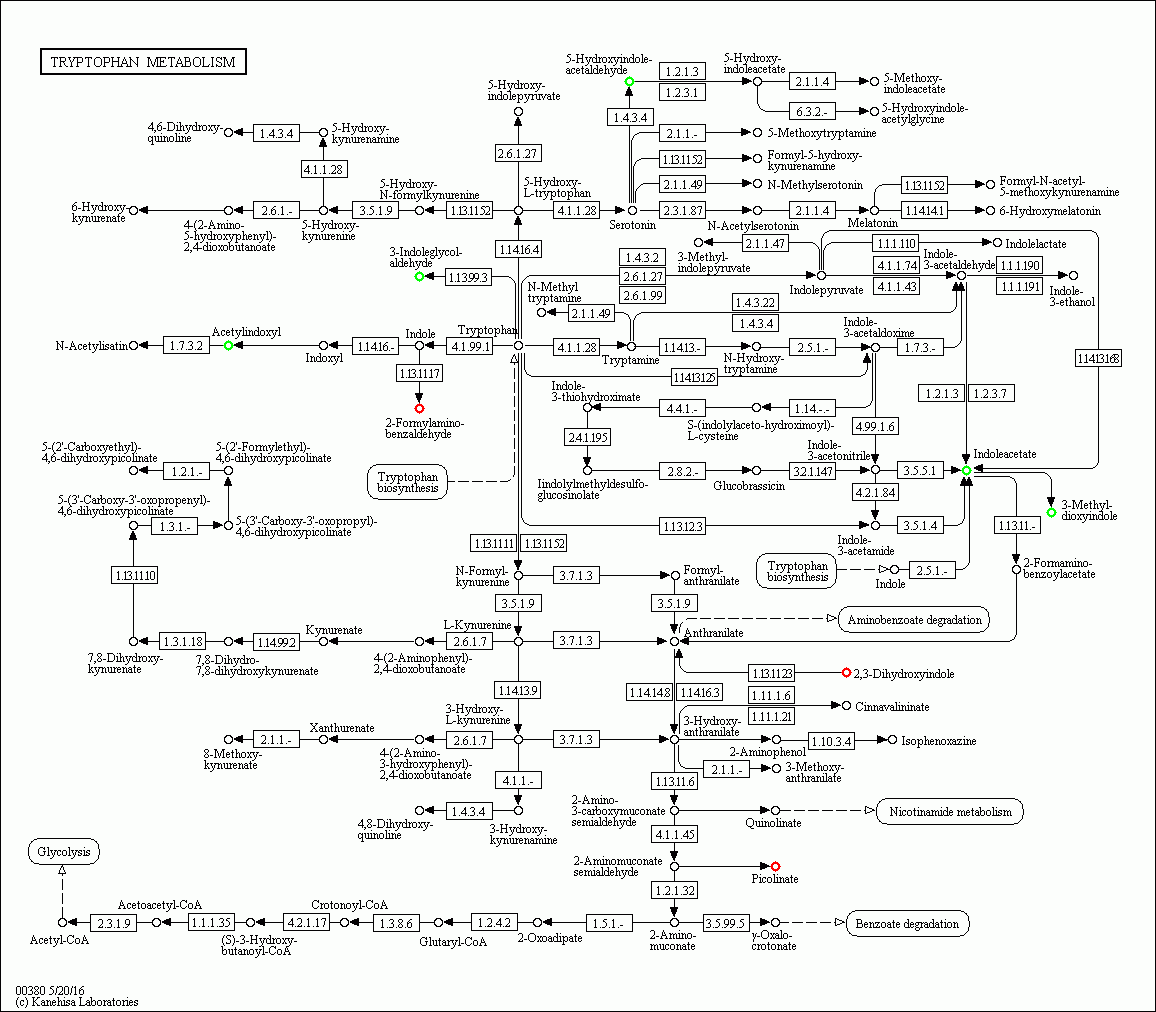


**Supplementary Figure 1**. **Map of tryptophan metabolism.** The red and green dots indicate the positions of different metabolites involved in this experiment. Red indicated upregulated and gene indicated downregulated.


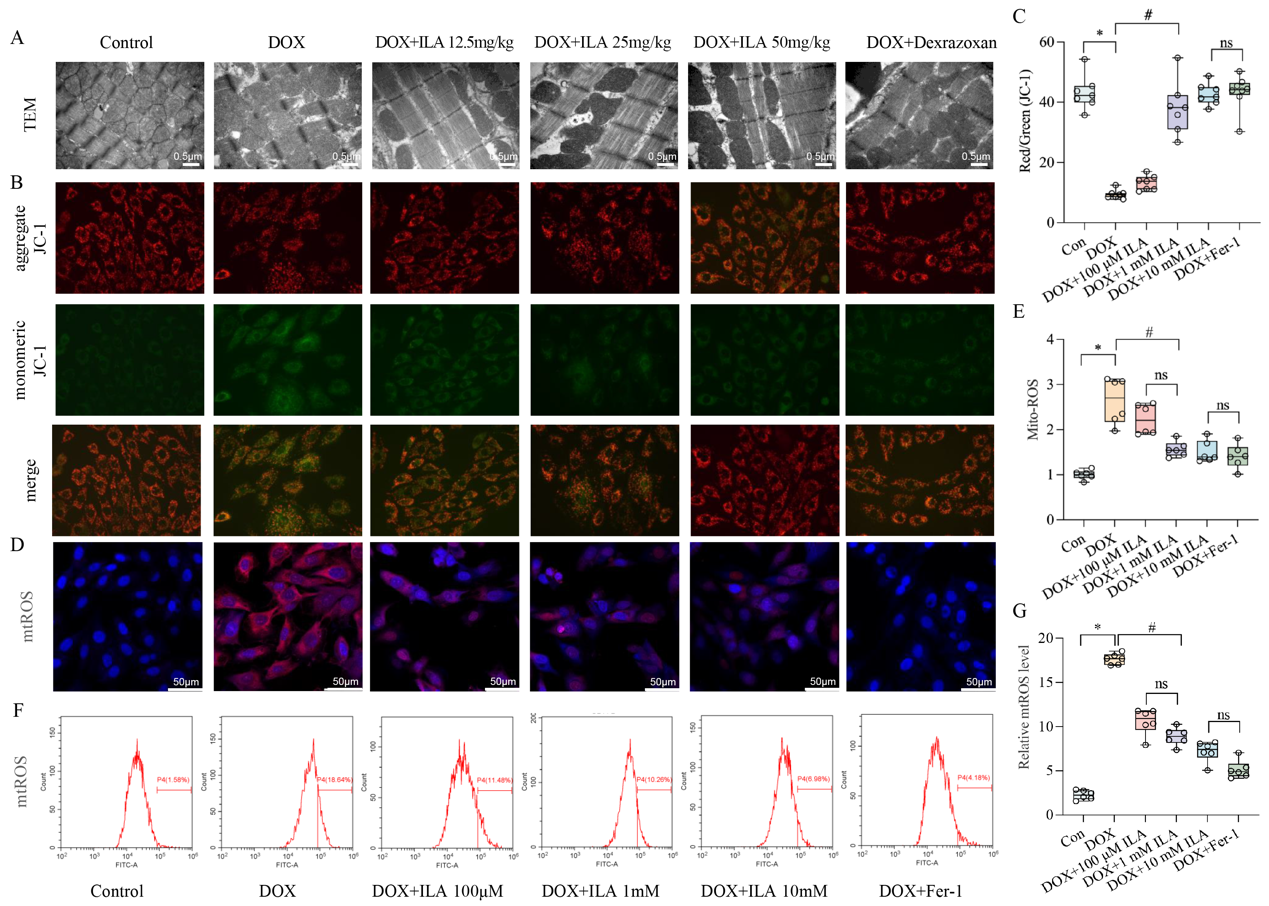


**Supplementary Figure 2**. **Indole-3-lactic acid (ILA) inhibits doxorubicin (DOX) induced mitochondrial oxidative stress.** (A) Transmission electron microscope was used to evaluate the changes of mitochondrial structural in mice from indicated groups. scale bar=0.5 μm. (B) Representative microscopy images of H9C2 cells that were stained with JC-1 dye, scale bar=50 μm. (C) Quantification of mitochondrial membrane potential of H9C2 cells via JC-1 monomer/JC-1 aggregates in (B). (D) Fluorescent images of H9C2 cells that were stained with Mito-Tracker Red, scale bar=50 μm. (E) Quantification of mtROS via MitoROS™ 580 intensity in (D). (F-G) Detection and quantification of cellular mtROS levels using flow cytometry.


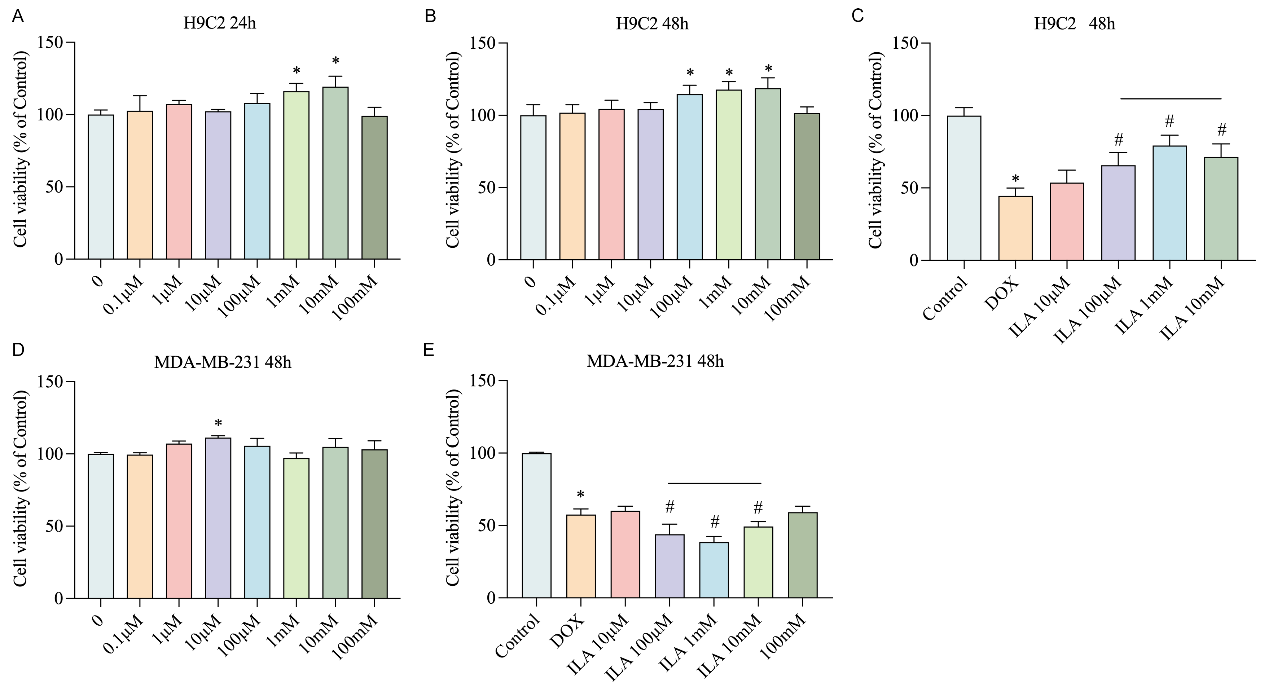


**Supplementary Figure 3**. **ILA inhibits doxorubicin (DOX)-induced death of cardiomyocytes.** Cell viability of H9c2 cells with a series of concentration of ILA treatment, for (A) 24h and (B) 48h. (C) 100μM, 1mM and 10mM ILA increased the viability of H9c2 cells with 1 μM DOX treatment. The values are presented as mean ± SD of three independent experiments. (D) Effect of ILA on DOX-induced death of MDA-MB-231 cells. (E) MDA-MB-231 cells were pretreated with ILA for 24 h. Then, 1 μM DOX was added for another 48 h, and cell viability was measured. *P<0.05 vs. Control; # P<0.05 vs. DOX.


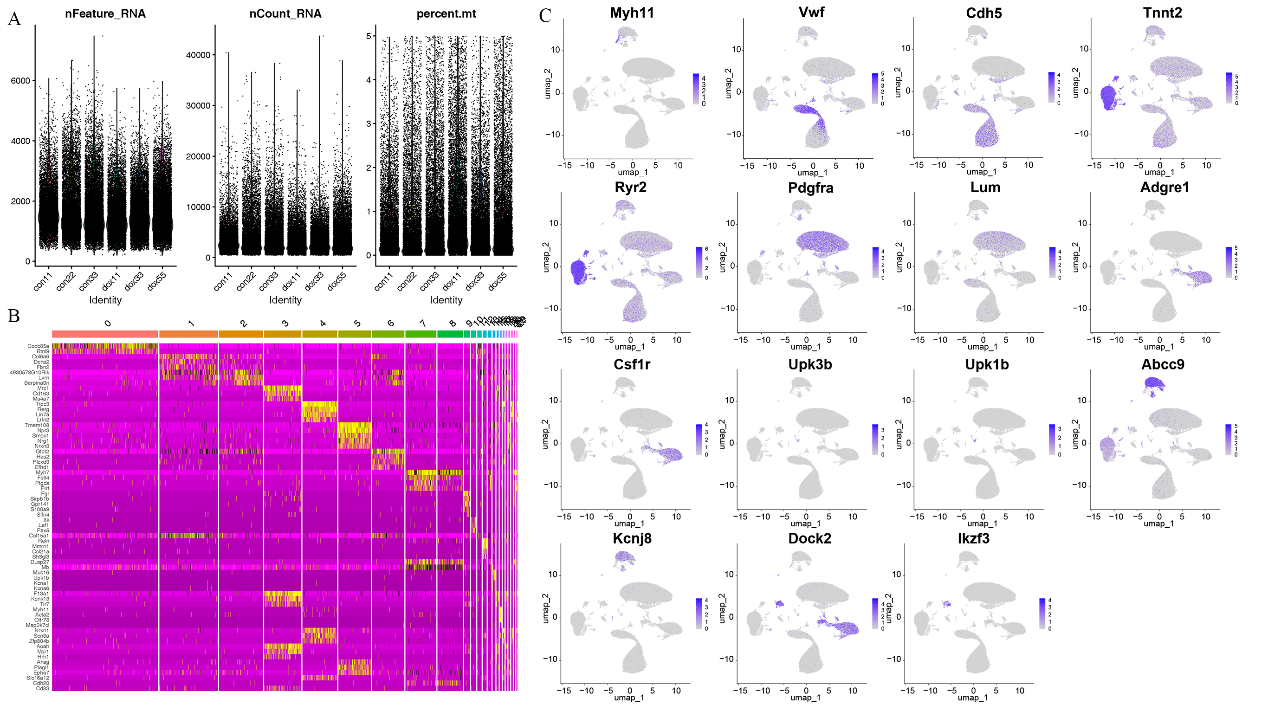


**Supplementary Figure 4**. Single nucleus RNA-sequencing data of doxorubicin-treated mice (n=3). (A) Quality control and sequencing information for six heart tissue. (B) Hierarchical clustering reveals the genes that define the identity of 23 cell cluster. (C) Feature plots of marker genes for cardiomyocytes (Tnnt2 and Ryr233), fibroblasts (Pdgfra, and Lum34), endothelial cells (Vwf and Cdh534), macrophage (Ikzf1, Csf1r, and Adgre135), smooth muscle cells (Myh11), pericardial cell (Upk1b and Upk3b34), pericytes (Kcnj8 and Abcc936), and B cell/T cell (Ikzf3 and Ikzf137).


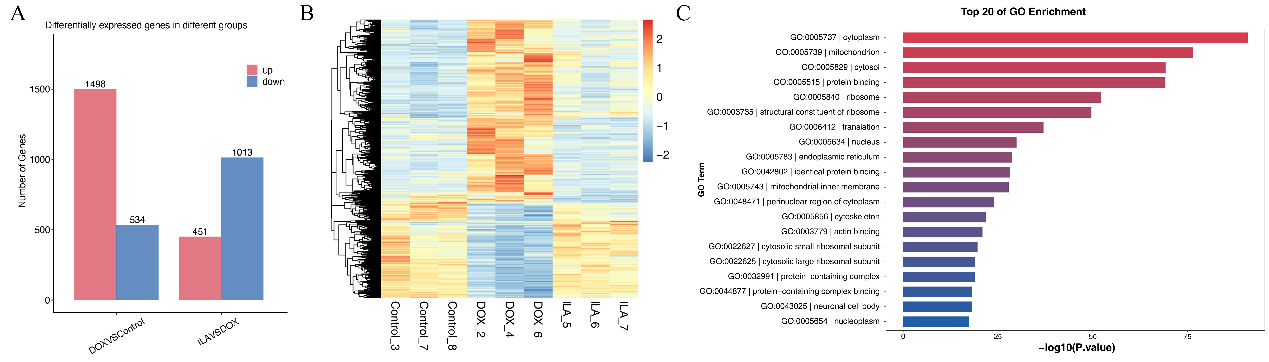


**Supplementary Figure 5**. RNA sequencing analysis of Indole-3-lactic Acid (ILA) treated mice. (A) Histogram of differentially expressed genes in the indicated groups (n = 3 for each group), by RNA sequencing. (B) Heatmap illustrating the z-score derived from transcripts per million (TPM) values of significantly modulated genes across the mice from the control, Doxorubicin and Doxorubicin+25mg/kg ILA group (n=3). (C) GO enrichment analysis of the top 998 differentially expressed genes.


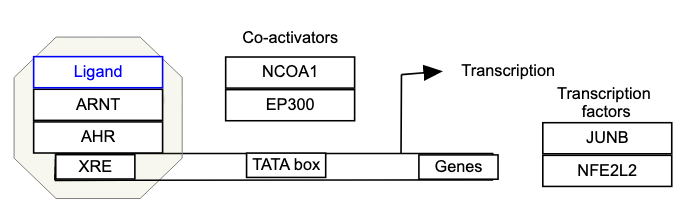


**Supplementary Figure 6**. **Prediction the influence of AhR on Nrf2 transcription online (www.wikipathways.org).**

**
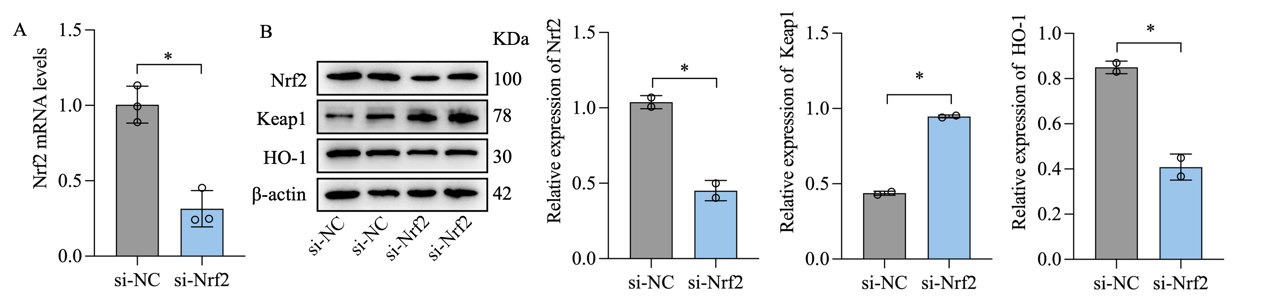
**

**Supplementary Figure 7**. **Inhibition of Nrf2 in H9C2 cells using specific siRNA.** (A) RT-qPCR assay was used to validate the knockdown of Nrf2 by transfection of Nrf2 siRNA. (B) Western blot showed the expression changes of Nrf2, Keap1 and HO-1 protein expression after inhibition of Nrf2 in H9C2 cells.


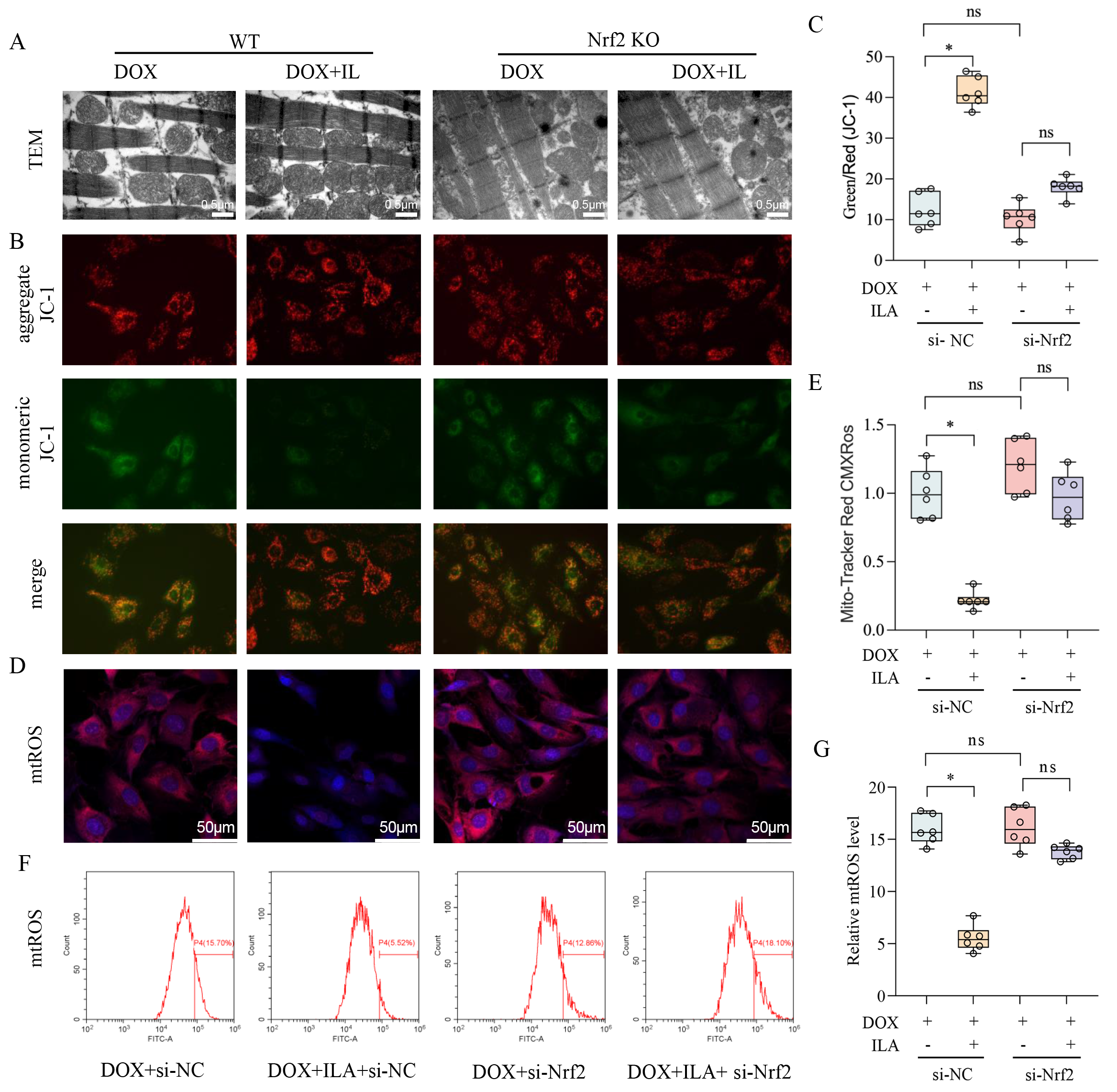


**Supplementary Figure 8**. **Indole-3-lactic acid (ILA) inhibits doxorubicin (DOX) induced mitochondrial oxidative stress via a Nrf2 dependent way.** (A) Transmission electron microscope was used to evaluate the changes of mitochondrial structural in Nrf2 knockout mice with or without DOX and ILA treatment. scale bar=0.5 μm. (B) Representative microscopy images of H9C2 cells that were stained with JC-1 dye, scale bar = 50 μm. (C) Quantification of mitochondrial membrane potential of Nrf2 silencing H9C2 cells via JC-1 monomer/JC-1 aggregates in (B). (D) Fluorescent images of H9C2 cells that were stained with Mito-Tracker Red, scale bar = 50 μm. (E) Quantification of mtROS via MitoROS™ 580 intensity in (D). (F-G) Detection and quantification of cellular mtROS levels using flow cytometry.
